# Gene and pathway level analyses of germline DNA-repair gene variants and prostate cancer susceptibility using the iCOGS-genotyping array

**DOI:** 10.1038/bjc.2016.50

**Published:** 2016-03-10

**Authors:** Edward J Saunders, Tokhir Dadaev, Daniel A Leongamornlert, Ali Amin Al Olama, Sara Benlloch, Graham G Giles, Fredrik Wiklund, Henrik Grönberg, Christopher A Haiman, Johanna Schleutker, Børge G Nordestgaard, Ruth C Travis, David Neal, Nora Pasayan, Kay-Tee Khaw, Janet L Stanford, William J Blot, Stephen N Thibodeau, Christiane Maier, Adam S Kibel, Cezary Cybulski, Lisa Cannon-Albright, Hermann Brenner, Jong Y Park, Radka Kaneva, Jyotsna Batra, Manuel R Teixeira, Hardev Pandha, Koveela Govindasami, Ken Muir, The UK Genetic Prostate Cancer Study Collaborators, Z Abbasi, M Akhlil Abdul-Hamid, Paul D Abel, Paul H Abrams, Fawzi A Adab, Andrew Adamson, A Adeyoju, Naveed Afzal, Ernest K N Ahiaku, Munir Ahmed, Mohammed L Al Sudani, Christopher Alcock, Zulfiqar Ali, David J Almond, Roberto Alonzi, Amir S M Al-Samarraie, Al-Samerraie, Waleed Al-Singary, Al-Sudani, John Anderson, Steven Andrews, Henry Andrews, Iqbal Anjum, Ken Anson, Nicola A Anyamene, Ike Apakama, F Aparcia, J A A Archbold, D Ash, Richard F U Ashford, A Azzabi, David Badenoch, Amit Bahl, M J Bailey, Karen Bailey, Andrew J Ball, G Banerjee, N Barber, Jim Barber, Baria, Douglas G Barnes, J Bashir, Pradip Basu, Christopher A Bates, N A Bax, D Baxter-Smith, Amar Bdesha, Christopher J M Beacock, Ronald P Beaney, Ralph Beard, John D Beatty, Rupert Beck, Gail Beese, Sharon Beesley, C Richard W Bell, James Bellringer, Richard Benson, Beresford, Christopher R A Bevis, Rajanee Bhana, S Bhanot, A Bhatnagar, R I Bhatt, Brian Birch, Alison Birtle, M Bishop, C Shekhar Biyani, A R E Blacklock, Rosemary Blades, Peter Bliss, David J Bloomfield, S Boddy, C M Booth, Pradeep Bose, Michael C Bott, David Bottomley, Nigel R Boucher, J Bowen, Mark Bower, W G Bowsher, P J R Boyd, F James Bramble, Simon F Brewster, Tim Briggs, Cathryn Brock, Sue Brock, Stephen Bromage, Richard Brough, Richard Brown, Stephen Brown, Richard Brown, Tony J Browning, N Bryan, Neil A Burgess, Nicholas Burns-Cox, Paul C Butterworth, D Cahill, P S Callaghan, John Calleary, M Calleja, Frances Calman, Philip Camilleri, Alister Campbell, Andrea Cannon, Dawn M Carnell, T W Carr, Simon Carter, Charles J M Carter, Adam C Carter, Bruce M Castle, David Chadwick, Rohit Chahal, P Chakraborti, Chappell, C Charig, Anula D Chetiyawardana, Christopher Chilton, F I Chinegwundoh, Irene Chong, Ananya Choudhury, Wai-Man Chow, Timothy J Christmas, Mark J Churn, Noel W Clarke, Jorge Clavijo-Eisele, M Coe, N P Cohen, C Coker, Trevor Cole, David J Cole, O Cole, Gerald Collins, Matthew Collinson, I Conn, C Connell, Audrey Cook, Peter Cooke, Graeme Cooksey, L Coombs, Robert F Copland, Andrew J Cornaby, P A Cornford, Corolis, John Corr, C B Costello, N Coull, Richard Cowan, Robert Cox, C Coyle, Jeremy Crew, John C Crisp, W Cross, W Cross, Dorthe Cruger, Malcolm Crundwell, Cummings, Nazeer Dahar, Francis N Daniel, J Darrad, Pallon Daruwala, Gautam Das, Shibendra Datta, S Davidson, Joseph Davies, Owen W Davison, Guy Dawkins, Chris Dawson, Alan R De Bolla, David Dearnaley, Ken M Desai, George P Deutsch, John Dick, Andrew J Dickinson, Jeanette Dickson, Michael Dinneen, Sanjay Dixit, H Jane Dobbs, A Doble, David Dodds, Alan Doherty, P Donaldson, M Dooldeniya, S Fiona Douglas, Drake, Gill M Duchesne, Peter Duffy, Michael Dunn, W D Dunsmuir, Sajid K Durrani, Alan C Eaton, Diane Eccles, B Eddy, C D Eden, J Edwards, Jeremy Elkabir, P Tony Elliott, B W Ellis, R Ellis, A El-Modir, Andrew W S Elves, Christine Elwell, Mark Emberton, Louise Emmerson, Roland C D England, R D Errington, D Gareth Evans, Alison Falconer, Derek Fawcett, C Featherston, Carolyn J Featherstone, Jeremy Feggetter, C Ferguson, D Fermont, Michael Ferro, Matthew Fletcher, A Folkes, Trevor F Ford, Paul W Foster, Kevin N Franks, Olivera Frim, Joanna Gale, Christopher Gallegos, James S Gelister, Ghana, Stephanie Gibbs, Hugh Gilbert, David Gillatt, John Glaholm, Jonathan M Glass, James Glenister, Thomas D Goode, E M Gordon, Richard L Gower, John Graham, Damian Green, Jonathan Greenland, Robert Grieve, Thomas R L Griffiths, Sandy Gujral, Nishi Gupta, Riza Murat Gurun, Peter J Guy, Neil Haldar, N Halder, F C Hamdy, C Hamilton, John Hammonds, S J Hampson, Damien C Hanbury, P D John Hardman, Stephen J Harland, John M Harney, Peter Harper, Sarah Harris, D Harris, G S M Harrison, D R Harriss, N Harvey-Hills, Simon Hawkyard, Catherine M Heath, Michael Hehir, Giles O Hellawell, David Hendry, Mike Henley, Ann Henry, John Hetherington, Tamas Hickish, James A Hicks, Serena Hilman, Richard Hindley, John R Hindmarsh, John Hines, M Hingorani, Edwin T S Ho, Shirley Hodgson, U Hoffman, David Holden, A Hollingdale, Graham W Hollins, Simon A V Holmes, Gail Horan, Alan Horwich, Peter Hoskin, Graham P Howell, D Hrouda, Robert Huddart, Liz Hudson, Rob Hughes, Michael Hughes, Owen Hughes, Caroline Humber, John W Iacovou, A Ibrahim, John A Inglis, Stuart Irving, C Irwin, Louise Izatt, Victor Izegbu, Basharat Jameel, Michael J James, N James, R Lester James, Pradip Javle, P Jenkins, Sameer Jhavar, Gareth Jones, Chris R Jones, David A Jones, J Joseph, Shelagh Joss, Amir Kaisary, Alexandre L Kaliski, G Kapur, O Karim, Stephen J Karp, F X Keeley, Anand R Kelkar, J P Kelleher, John Kelly, Sue Kenwrick, F Khan, Vincent Khoo, Rachel M Kimber, R Kinder, Roger S Kirby, David Kirk, Peter Kirkbride, Magdi M Kirollos, Roger Kockelbergh, Philip C W C Koenig, Gordon G Kooiman, O Koreich, Anthony Koupparis, Mohamed Kourah, Sigurd Kraus, Magda L Kujawa, Ravi Kulkarni, M Kumar, Ian H Kunkler, H Kynaston, Katherine L Lachlan, Robert Laing, Fiona Lalloo, M Lancashire, Stephen E M Langley, Marc Laniado, T R Larner, Maurice W Lau, W T Lawrence, Anne Lawson, Pieter J Le Roux, Mary Leader, J O Lee, L Lee, A Lee, R John Lemburger, Priscilla Leone, Jason Lester, Hing Leung, J Lewis, D Christopher Lewis, Thomas Liston, Jacqueline Livsey, S Lloyd, Imogen Locke, Richard Lodge, John Logue, Mark Longmuir, Malcolm G Lucas, C J Luscombe, Anna Lydon, Michael Lynch, Naing N K Lynn, James P A MacDermott, Ruaraidh P Macdonagh, Macdonald, Sanjeev Madaan, Kudingila R Madhava, Joseph Maguire, E R Maher, Rana Mahmood, Graeme H M Mair, Peter R Malone, Stephen A Mangar, Mark Mantle, I Mark, Robert Mason, M D Mason, Matanhelia, Shyam Matenhelia, Philip N Matthews, J McAleese, Donna McBride, Jonathan McFarlane, McGrath, Craig McIlhenny, Paul McInerney, Gregor McIntosh, F McKinna, Duncan McLaren, Esther McLarty, Rhona McMenemin, Alan McNeill, T A McNicholas, Robert N Meddings, A David Mee, Lucinda Melcher, Memon, Pravin Menzes, Marek Miller, Robert Mills, S Mitchell, Natasha Mithal, Anita Mitra, Gillian E Mobb, Leslie E F Moffat, Mokete, Julian Money-Kyrle, Bruce Montgomery, Martin P Moody, Roland Morley, Sean B Morris, Patrick Morrison, Diana Mort, Amir H Mostafid, Hanif Motiwala, Gulzar Mufti, Gordon Muir, Faiz Mumtaz, Michael Murphy, Keith W Murray, Alexandra Murray, Shirley Murrell, D Muthukumar, Harry Naerger, Siva Namasivayam, Vinod Nargund, Nawrocki, Donald Neilson, A Nethersell, Julian Barwell, Jacqueline C Newby, Hugh Newman, R Newton, Neil Oakley, P J O'Boyle, J O'Brien, Tim S O'Brien, H O'Donnell, Neil O'Donoghue, E O'Donoghue, Chris Ogden, Hemant Ohja, Tim Oliver, Eng K Ong, P O'Reilly, J S O'Rourke, David Osborn, Peter Ostler, Joe O'Sullivan, J Owen, Edward Palfrey, Miguel Panades, Niki Panakis, M Pancharatnam, Michalakis L Pantelides, U Panwar, Omi Parikh, Chris Parker, Christopher H Parker, Bohdan T Parys, Sarah Pascoe, Anup Patel, Joan Paterson, S Pathack, Jhumur Pati, Helen Patterson, Pattu, A Paul, Heather Payne, David Peake, I Pedley, A Pengelly, Amjad M Peracha, Matthew Perry, Raj Persad, John Peters, N H Philp, T Philp, Lisa M Pickering, Katharine Pigott, R Plail, P Nicholas Plowman, Richard D Pocock, A J Pope, Rick Popert, Tim Porter, John M Potter, Christopher Powell, Thomas B Powles, Krishna Prasad, Seshadri Sri Prasad, J W Prejbisz, Stephen Prescott, Andrew Protheroe, Khaver N Qureshi, Nigel Raby, Narasimhan Ragavan, Palaniappa G S Raju, Prakash B Ramachandra, R Raman, Abhay Rane, Julia Rankin, Y Rao, Hari L Ratan, Ramachandran Ravi, K Ravishankar, Read, Paul J Reddy, Peter R Rimington, Peter A Ritchie, J Trevor Roberts, Andrew Robertson, Angus Robinson, Anne C Robinson, Lee Q Robinson, Mark A Rochester, P B Rogers, Tomas P Rosenbaum, Neil Rothwell, Carl Rowbotham, Rowe, Kathryn Rowley, Deborah Ruddy, John Rundle, John M Russell, P G Ryan, A Sabharwal, Anand K Saggar, Ali Samanci, Vijay K Sangar, M F Saxby, Hartwig Schwaibold, John E Scoble, Christopher Scrase, Selim, Henry Sells, Krishna K Sethia, David C Shackley, Shaffer, Nihil Shah, D Shakespeare, Sue Shanley, Neerah K Sharma, Denise J Sheehan, Elizabeth Sherwin, Poh Lin Shum, LucySide, Norma Sidek, Karol Sikora, R Simcock, Andrew M Sinclair, Pravin Singh, M Siva, Michael F Smith, James Smith, Michael Sokal, Graham M Sole, Mark J Speakman, Alexander Spiers, Thiagarajan Sreenivasan, Narayanan N Srihari, Srinivasan, Rajagopalan Sriram, John N Staffurth, D Stewart, Andrew Stockdale, Mark A Stott, M J Stower, John R Strachan, Nicholas S A Stuart, Elaine Sugden, Duncan Summerton, Santhanam Sundar, S K Sundaram, Gokarakonda Suresh, Shabbir Susnerwala, Kuchibhotla S Swami, Stephanie J Symons, Isabel Syndikus, Saad Tahir, J Tanquay, John W Taylor, J W Taylor, T Terry, Robert J Thomas, Stephen A Thomas, Alan Thompson, Alastair H Thomson, A Thurston, Owen Tilsley, Stuart F Tindall, K Tipples, Tong, Hamid Toussi, Elizabeth W Toy, Richard C Trembath, David N Tulloch, Kevin J Turner, James Tweedle, C J Tyrell, N Umez-Eronini, Graeme H Urwin, Justin A Vale, Van As, Nicholas Van As, Subramaniam Vasanthan, Sean Vesey, Maria Vilarino-Varela, John Violet, Jaspal Virdi, Robert Wade, Katherine Waite, E M Walker, Roger Walker, David M A Wallace, Nicholas A Watkin, M E Watson, J H Waxman, Brian Waymont, Andrew Weaver, Ralph J Webb, Andrew Wedderburn, Paula Wells, G D Wemyss-Holden, P M T Weston, Duncan Wheatley, P Whelan, D Whillis, Adam D Wilde, Vicki Wiles, Marie Wilkins, John H Williams, Simon Williams, Michael Willis, Michael I Wills, Richard Wilson, J R Wilson, Mathias H Winkler, Marcus Wise, Simon Woodhams, C Woodhouse, Cathryn Woodward, Woolf, K A Woolfenden, Jane Worlding, Mark Wright, WYLIE, James P Wylie, Chris Wynne, Angelika Zang, A Zarkar, The UK ProtecT Study Collaborators, Angela Cox, Paul M. Brown, Anne George, Gemma Marsden, Athene Lane, Michael Davis, Prasad Bollina, Sue Bonnington, Lynne Bradshaw, James Catto, Debbie Cooper, Liz Down, Andrew Doble, Alan Doherty, Garrett Durkan, Emma Elliott, David Gillatt, Pippa Herbert, Peter Holding, Joanne Howson, Mandy Jones, Roger Kockelbergh, Rajeev Kumar, Howard Kynaston, Athene Lane, Teresa Lennon, Norma Lyons, Hing Leung, Malcolm Mason, Hilary Moody, Philip Powell, Alan Paul, Stephen Prescott, Derek Rosario, Patricia OSullivan, Pauline Thompson, Sarah Tidball, The PRACTICAL Consortium, Margaret Cook, Angela Morgan, Artitaya Lophatananon, Cyril Fisher, Malgorzata Tymrakiewicz, Michelle Guy, Rosemary Wilkinson, Sara Jugurnauth-Little, Steve Hazel, Melissa C Southey, Liesel M Fitzgerald, John Pedersen, John Hopper, Ami Karlsson, Carin Cavalli-Bjoerkman, Jan-Erik Johansson, Jan Adolfson, Markus Aly, Michael Broms, Paer Stattin, Brian E Henderson, Fredrick Schumacher, Anssi Auvinen, Kimmo Taari, Liisa Maeaettaenen, Paula Kujala, Teemu Murtola, Teuvo LJ Tammela, Tiina Wahlfors, Andreas Roder, Peter Iversen, Peter Klarskov, Sune F Nielsen, Tim J Key, Hans Wallinder, Sven Gustafsson, Jenny L Donovan, Freddie Hamdy, Angela Cox, Anne George, Athene Lane, Gemma Marsden, Michael Davis, Paul Brown, Paul Pharoah, Lisa B Signorello, Wei Zheng, Shannon K McDonnell, Daniel J Schaid, Liang Wang, Lori Tillmans, Shaun Riska, Thomas Schnoeller, Kathleen Herkommer, Manuel Luedeke, Walther Vogel, Dominika Wokolorczyk, Jan Lubiski, Wojciech Kluzniak, Katja Butterbach, Christa Stegmaier, Bernd Holleczek, Hui-Yi Lin, Hyun Park, Julio Pow-Sang, Thomas Sellers, Chavdar Slavov, Aleksandrina Vlahova, Atanaska Mitkova, Darina Kachakova, Elenko Popov, Svetlana Christova, Tihomir Dikov, Vanio Mitev, Allison Eckert, Amanda Spurdle, Angus Collins, Glenn Wood, Greg Malone, Judith A Clements, Kris Kerr, Megan Turner, Pamela Saunders, Peter Heathcote, Srilakshmi Srinivasan, Leire Moya, Trina Yeadon, Joana Santos, Carmen Jerónimo, Paula Paulo, Pedro Pinto, Rui Henrique, Sofia Maia, Agnieszka Michael, Andrzej Kierzek, Huihai Wu, Australian Prostate Cancer BioResource, Douglas F Easton, Rosalind A Eeles, Zsofia Kote-Jarai

**Affiliations:** 1 The Institute of Cancer Research & Royal Marsden NHS Foundation Trust, 123 Old Brompton Rd, London SW7 3RP, UK; 2 Centre for Cancer Genetic Epidemiology, Department of Public Health and Primary Care, University of Cambridge, Strangeways Laboratory, Worts Causeway, Cambridge CB1 8RN, UK; 3 Cancer Epidemiology Centre, The Cancer Council Victoria, 1 Rathdowne Street, Carlton Victoria, Australia; 4 Centre for Molecular, Environmental, Genetic and Analytic Epidemiology, The University of Melbourne 3053, Victoria, Australia; 5 Department of Medical Epidemiology and Biostatistics, Karolinska Institute, Stockholm 17177, Sweden; 6 Department of Preventive Medicine, Keck School of Medicine, University of Southern California & Norris Comprehensive Cancer Center, Los Angeles, CA 90089, USA; 7 Department of Medical Biochemistry and Genetics, University of Turku, Turku, Finland; 8 Institute of Biomedical Technology and BioMediTech, University of Tampere and FimLab Laboratories, Tampere 33520, Finland; 9 Department of Clinical Biochemistry, Herlev and Gentofte Hospital, Copenhagen University Hospital, Herlev Ringvej 75 DK-2730, Herlev, Denmark; 10 Cancer Epidemiology Unit, Nuffield Department of Population Health, University of Oxford, Oxford OX3 7LF, UK; 11 Surgical Oncology (Uro-Oncology: S4), University of Cambridge, Addenbrooke's Hospital, Hills Road, Cambridge & Cancer Research UK Cambridge Research Institute, Li Ka Shing Centre, Cambridge CB2 2QQ, UK; 12 University College London, Department of Applied Health Research, 1-19 Torrington Place, London WC1E 7HB, UK; 13 Cambridge Institute of Public Health, University of Cambridge, Forvie Site, Robinson Way, Cambridge CB2 0SR, UK; 14 Department of Epidemiology, School of Public Health, University of Washington & Division of Public Health Sciences, Fred Hutchinson Cancer Research Center, Seattle, WA, USA; 15 International Epidemiology Institute, 1455 Research Blvd., Suite 550, Rockville MD 20850, USA; 16 Mayo Clinic, Rochester, MN 55905, USA; 17 Institute of Human Genetics, University Hospital Ulm, Ulm 89075, Germany; 18 Division of Urologic Surgery, Brigham and Women's Hospital, Dana-Farber Cancer Institute, 45 Francis Street- ASB II-3 Boston, MA, 02245, USA; 19 International Hereditary Cancer Center, Department of Genetics and Pathology, Pomeranian Medical University, Szczecin 70-115, Poland; 20 Division of Genetic Epidemiology, Department of Medicine, University of Utah School of Medicine & George E. Wahlen Department of Veterans Affairs Medical Center, Salt Lake City, UT 84132, USA; 21 Division of Clinical Epidemiology and Aging Research, German Cancer Research Center (DKFZ), Heidelberg & Division of Preventive Oncology, German Cancer Research Center (DKFZ) and National Center for Tumor Diseases (NCT), Heidelberg & German Cancer Consortium (DKTK), German Cancer Research Center (DKFZ), Heidelberg, Germany; 22 Department of Cancer Epidemiology, H. Lee Moffitt Cancer Center, 12902 Magnolia Drive, Tampa, FL 33612, USA; 23 Molecular Medicine Center and Department of Medical Chemistry and Biochemistry, Medical University - Sofia, 2 Zdrave Street, Sofia 1431, Bulgaria; 24 Australian Prostate Cancer Research Centre-Qld, Institute of Health and Biomedical Innovation & School of Biomedical Science, Queensland University of Technology, Brisbane 4102, Australia; 25 Biomedical Sciences Institute (ICBAS), Porto University, Porto, Portugal; 26 Department of Genetics, Portuguese Oncology Institute, Porto, Portugal 4200-072, Portugal; 27 The University of Surrey, Guildford, Surrey GU2 7XH, UK; 28 Warwick Medical School, University of Warwick, Coventry CV4 7AL, UK

**Keywords:** DNA repair, prostate cancer, genome-wide association study, GWAS, iCOGS

## Abstract

**Background:**

Germline mutations within DNA-repair genes are implicated in susceptibility to multiple forms of cancer. For prostate cancer (PrCa), rare mutations in *BRCA2* and *BRCA1* give rise to moderately elevated risk, whereas two of ∼100 common, low-penetrance PrCa susceptibility variants identified so far by genome-wide association studies implicate *RAD51B* and *RAD23B*.

**Methods:**

Genotype data from the iCOGS array were imputed to the 1000 genomes phase 3 reference panel for 21 780 PrCa cases and 21 727 controls from the Prostate Cancer Association Group to Investigate Cancer Associated Alterations in the Genome (PRACTICAL) consortium. We subsequently performed single variant, gene and pathway-level analyses using 81 303 SNPs within 20 Kb of a panel of 179 DNA-repair genes.

**Results:**

Single SNP analyses identified only the previously reported association with *RAD51B*. Gene-level analyses using the SKAT-C test from the SNP-set (Sequence) Kernel Association Test (SKAT) identified a significant association with PrCa for *MSH5*. Pathway-level analyses suggested a possible role for the translesion synthesis pathway in PrCa risk and Homologous recombination/Fanconi Anaemia pathway for PrCa aggressiveness, even though after adjustment for multiple testing these did not remain significant.

**Conclusions:**

*MSH5* is a novel candidate gene warranting additional follow-up as a prospective PrCa-risk locus. *MSH5* has previously been reported as a pleiotropic susceptibility locus for lung, colorectal and serous ovarian cancers.

Prostate Cancer (PrCa) is the most frequently diagnosed cancer among men in developed countries and despite high survival rates also one of the highest for mortality (Cancer Research UK, 2014; Quaresma *et al*, 2015). However, as the majority of prostate neoplasms develop extremely slowly, many do not require clinical intervention, which coupled with the low specificity of the prostate-specific antigen test for clinically relevant forms of the disease could potentially lead to considerable over-diagnosis and overtreatment of patients for relatively modest reductions in mortality (Ilic *et al*, 2013). In conjunction with the establishment of improved biomarkers for lethal PrCa, the identification of individuals at greater risk of developing prostate tumours that require clinical intervention would also help inform more targeted and appropriate application of treatment. The heritability of PrCa is believed to be the highest of all the common forms of cancer (Hjelmborg *et al*, 2014). This is consistent with observations from genome-wide association studies (GWAS), which have to date identified >100 low-penetrance susceptibility variants for PrCa, two of which implicate the DNA-repair genes *RAD51B* and *RAD23B* (Xu *et al*, 2012; Al Olama *et al*, 2014; Eeles *et al*, 2014; Amin Al Olama *et al*, 2015). In addition, rare germline mutations in a small number of genes have been reported, with varying degrees of evidence, as potentially conferring greater risks of PrCa, including the DNA-repair genes *ATM*, *BRCA1*, *BRCA2*, *BRIP1*, *CHEK2* and *NBN* (Dong *et al*, 2003; Kote-Jarai *et al*, 2009, 2011; Leongamornlert *et al*, 2012, 2014; Robinson *et al*, 2015). Recently, increasing evidence has demonstrated that these germline DNA-repair gene mutation carriers are at increased likelihood of experiencing advanced disease, metastatic spread and poorer survival outcome; yet these mutations also hold promise as potentially clinically actionable and responsive to targeted treatments (Castro *et al*, 2013; Cybulski *et al*, 2013; Leongamornlert *et al*, 2014; Robinson *et al*, 2015). In spite of these discoveries, the majority of the excess familial risk of PrCa still remains to be explained (Attard *et al*, 2015), with the contribution of DNA-repair gene variants identified to date making them attractive candidates for further investigation. In this study, using data from the iCOGS project imputed to the 1000 Genomes Phase 3 reference panel, we have analysed a large panel of DNA-repair gene variants for 21 780 PrCa cases and 21 727 controls of European ancestry from the Prostate Cancer Association Group to Investigate Cancer Associated Alterations in the Genome (PRACTICAL) Consortium (Eeles *et al*, 2013). Analyses were performed at single variant, gene and pathway levels to maximise the power to detect putative associations with lower frequency variants or those with modest effect sizes.

## Materials and methods

### Samples

Samples for the iCOGS study were drawn from 25 studies participating in the PRACTICAL Consortium. The majority of studies were population-based or hospital-based case–control studies, or nested case–control studies; some studies selected samples by age or oversampled for cases with a family history of prostate cancer. Further information regarding the samples from the PRACTICAL Consortium included on the iCOGS array may be found within the original publication (Eeles *et al*, 2013). Analyses for DNA-repair gene variants were restricted to samples of European ancestry. In total, genotype data for 21 780 PrCa cases and 21 727 matched controls were available after quality control.

### Genotyping and imputation

Genotyping was performed as part of the iCOGS project. This utilised a custom genotyping array designed in collaboration between the PRACTICAL, BCAC (Breast Cancer Association Consortium), OCAC (Ovarian Cancer Association Consortium) and CIMBA (Consortium of Investigators of Modifiers of BRCA1/2) consortia. Detailed information about the design, genotyping and quality control procedures for iCOGS can be found within the original publication (Eeles *et al*, 2013). In total 211 155 SNPs were genotyped on the iCOGS array, of which 3510 were situated within our defined DNA-repair gene regions. Imputation of the iCOGS PRACTICAL data was performed based on sequence data for 2504 samples from the 1000 Genomes phase 3 reference panel (IMPUTE2 haplotype panel, October 2014 release; https://mathgen.stats.ox.ac.uk/impute/1000GP%20Phase%203%20haplotypes%206%20October%202014.html) using SHAPEIT (v2 r778) and IMPUTE v2.3.1 in 588 chunks with a median size of 5 Mb (Howie *et al*, 2009; Delaneau *et al*, 2013). Imputed data for variants with INFO scores ⩾0.3 and MAF >0.001 were included in these analyses, which retained a total of 81 303 variants within the studied DNA-repair gene regions.

### Gene/region selection

We identified a total of 179 genes with a core function in DNA-damage repair from the literature that intersected imputed iCOGS genotype data. We annotated DNA-repair genes to a single primary DNA-repair pathway according to previous curations (Wood *et al*, 2005; Kang *et al*, 2012). The genes analysed in this study represent the pathways Homologous recombination/Fanconi Anaemia signalling network (HR/FA), base excision repair (BER), non-homologous end joining (NHEJ), mismatch repair (MMR), nucleotide excision repair (NER), translesion synthesis (TLS), ATM signalling (ATM), RECQ helicase family (RECQ), cross-link repair (XLR), and other miscellaneous DNA-repair genes with functions including endonuclease/exonuclease activity and modification of chromatin structure (Other). Gene coordinates were assigned according to GENCODE release 19 (GRCh37.p13), with a 20-kb flank added to define the study region for each gene, in order to focus primarily on capturing gene and promoter centric variation over that within regulatory elements, which can be located at variable and potentially relatively large distances from the gene itself. Variants were annotated using wANNOVAR to facilitate designation as coding, intronic, UTR, splice and intergenic (Wang *et al*, 2010; Chang and Wang, 2012). A full list of the DNA-repair genes analysed in this study, their pathway annotations, region coordinates and the number of typed and imputed variants available is included in Supplementary Table 1.

### Statistical analyses

Analyses were adjusted for study groups and the first eight principal components. For single-SNP analyses the genome-wide significance threshold was employed (*P*<5 × 10^−8^), whereas for gene and pathway level tests the Bonferroni correction was used to determine multiple testing adjusted significance thresholds (gene *P*<2.7 × 10^−4^, pathway *P*<5.56 × 10^−3^).

All analyses were carried out using R. For single-SNP analyses, per allele odds ratios were estimated using logistic regression. SKAT tests were performed using the SKAT package for R (http://CRAN.R-project.org/package=SKAT). We used the SKAT-O and SKAT-C tests for optimal analyses of the combined effect of multiple rare variants and common and rare variants, respectively (Wu *et al*, 2011; Lee *et al*, 2012; Ionita-Laza *et al*, 2013). Tests were conducted using default parameters and a common/rare cutoff threshold of MAF=0.01 for the SKAT-C test. StepAIC and SKAT leave one out were used to further interrogate the significant SKAT signal at the *MSH5* gene for the individual variants that best described the signal.

Analyses for low-grade *vs* high-grade PrCa were carried out based on two clinical criteria. For stringent comparison of non-aggressive and aggressive PrCa, we defined NCCN stage 1 patients as non-aggressive PrCa and individuals with metastatic disease (M^+^) or nodal spread (N^+^) as aggressive (395 NCCN1 *vs* 1391 M^+^/N^+^), whereas to enhance the sample panel available for this analysis we also compared patients with Gleason Stage (GS) ⩽6 against those with GS ⩾8 disease (9626 GS ⩽6 *vs* 2776 GS ⩾8).

## Results

Using genotype data from the iCOGS study imputed to the 1000 genomes phase 3 reference panel we analysed 81 303 SNPs within a 20-kb flanking region of 179 genes with a core function in DNA-damage repair (Supplementary Table 1). Rare and uncommon variants represented a substantial proportion of the data set, with 29 503 variants of MAF ⩽1%, 16 689 with MAF 1–5% and 35 111 with MAF >5% (Supplementary Figure 1a). Variants were categorised as SNPs, insertions and deletions, annotated using wANNOVAR (Wang *et al*, 2010; Chang and Wang, 2012), and classified into five categories; coding, UTR, splice, intronic and intergenic. Variants available for this analysis were predominantly situated within non-coding (intronic or intergenic) regions, with 3943 variants annotated as coding, splice or UTR in total, whereas most were single-base substitutions, with 3914 insertions and 5576 deletions, respectively. All of the insertion and deletion variants were imputed, with the vast majority located within non-coding regions (Supplementary Figure 1b–d, Supplementary Table 2). All analyses were adjusted for study population and the first eight principal components. For single-variant level analyses the genome-wide significance threshold (*P*<5 × 10^−8^) was used to determine significantly associated variants, whereas for gene and pathway level analyses the significance threshold was defined according to the Bonferroni correction (gene *P*<2.7 × 10^−4^, pathway *P*<5.56 × 10^−3^).

**Figure 1:**
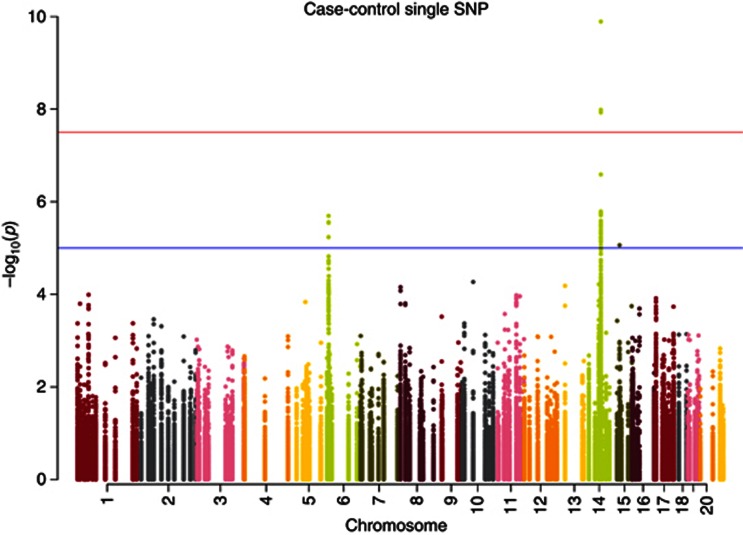
**Single SNP case–control Manhattan Plot.** In total, 81 303 SNPs from 179 DNA-repair genes were analysed for association with PrCa. Only the previously reported association within the *RAD51B* gene was identified, with suggestive, non-significant association peaks observed at a small number of other loci.

Single-variant analysis for association of DNA-repair gene variants with PrCa identified only the previously reported association with *RAD51B* at Chr14q24 (rs371311594, *P*=1.29 × 10^−10^). Several other gene loci showed suggestive association peaks; however, no other variants were within one order of magnitude of genome-wide significance (Figure 1, Supplementary Table 3).

**Figure 2:**
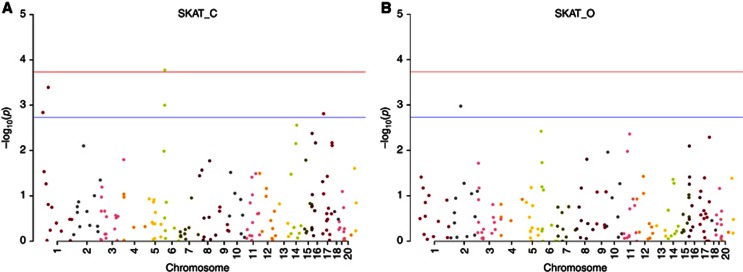
**Case–control Manhattan Plots for the 179 DNA-repair genes analysed by SKAT.** (**A**) A significant association was observed for the *MSH5* gene using the SKAT-C test that examines the combined effect of common and rare variants. (**B**) No significant association was detected for any gene under the SKAT-O test that primarily focuses on rare variant association testing.

We observed evidence for modest inflation within our association data (*λ*=1.105); nonetheless, departure from the null was apparent towards the extremity of the *P*-value distribution and this persisted to a more modest extent even after the *RAD51B* region was excluded (Supplementary Figure 2). We subsequently performed gene level association tests, in an attempt to ascertain whether additional putative PrCa-risk signals might be present among the genes within which no individual variant achieved significance after adjustment for multiple testing, arising through a cumulative effect of several low MAF or low penetrance variants. We performed two gene-level association tests using the SKAT; SKAT-C, which is optimised for combined testing of rare and common variants and SKAT-O, which attempts to maximise power for rare variant testing (Lee *et al*, 2012; Ionita-Laza *et al*, 2013). Gene-level analysis identified a novel significant association with the *MSH5* gene using the SKAT-C test (Chr6p21; *P*=1.68 × 10^−4^) (Figure 2, Supplementary Table 4). We used stepAIC and leave one out for SKAT to further interrogate the *MSH5* data for the individual variants that best explain the signal. This test selected three variants at the *MSH5* locus, rs61036903 (known as 6 : 31713892 within the reference panel) intronic within the gene and two variants 10-kb downstream within an adjacent gene *VWA7*, rs805825 and rs185333600. These were all among the top-ranking variants in the single-SNP analysis (rs61036903: MAF=0.14, OR 0.92, *P*=8.06 × 10^−5^; rs805825: MAF=0.45, OR 0.94, *P*=4.05 × 10^−5^; rs185333600: MAF=0.003, OR 1.57, *P*=6.83 × 10^−4^).

We subsequently examined the iCOGS data set at the pathway level under the SKAT test to supplement the gene-level analyses. We again used the Bonferroni correction to define the significance threshold (pathway *P*<5.56 × 10^−3^). No pathway achieved significance at this threshold, with suggestive associations under the SKAT-O test observed with the translesion synthesis pathway (*P*=6.18 × 10^−3^) and mismatch-repair pathway (*P*=0.056).

Variants within the coding sequence of DNA-repair genes could be more likely to influence PrCa risk than those in non-coding regions. We therefore performed an additional SKAT test to assess whether the coding DNA-repair gene variants available for this study, when collapsed as a single entity, could stratify case and control status. We observed a significant association when using the SKAT-C test (*P*=0.003), which suggests that variants that affect the coding sequence of genes participating in DNA-repair processes contribute to PrCa risk. We attempted to further elaborate upon this finding by analysing coding variation within each pathway separately. Despite relatively modest numbers of coding variants available within each pathway, we continued to observe suggestive associations under the SKAT-O test for the translesion synthesis pathway (*P*=0.026) and mismatch-repair pathway (*P*=0.055), in addition to the HR/FA pathway under the SKAT-C test (*P*=0.011).

To complement the tests designed to identify potential PrCa susceptibility variants and genes, we also performed case–case analyses to investigate whether individual or cumulative germline DNA-repair gene and pathway variants in the iCOGS imputed data set correlated with phenotypic characteristics of more aggressive PrCa. This analysis was limited by lack of complete phenotypic data for all patients within the iCOGS sample set and low numbers of samples within individual phenotypic subgroups; therefore, we utilised two separate criteria to define aggressive and non-aggressive disease. For a stringent comparison of non-aggressive and aggressive PrCa, we analysed NCCN stage 1 patients against individuals with metastatic disease (M^+^) or nodal spread (N^+^) (395 NCCN1 *vs* 1391 M^+^/N^+^), whereas to maximise the number of samples available we also compared patients with GS ⩽6 disease against those with GS ⩾8 (9626 GS ⩽6 *vs* 2776 GS ⩾8). No significant associations with aggressive PrCa were identified at either the variant or gene levels for either of the phenotypic criteria tested. (Supplementary Figure 3, Supplementary Table 5). When we examined PrCa aggressiveness at the pathway level, we observed associations at *P*<0.05 for the HR/FA pathway under both tests for the GS ⩽6 *vs* GS ⩾8 phenotype cohort (SKAT-C *P*=0.011, SKAT-O *P*=0.040). This pathway was also the highest ranked for the NCCN1 *vs* M^+^/N^+^ phenotype cohort under the SKAT-C test (*P*=0.052). When these analyses were restricted to only coding variants, an association at *P*<0.05 remained for the HR/FA pathway for the NCCN1 *vs* M^+^/N^+^ cohort and the SKAT-O test (*P*=0.021). These suggestive associations were not however significant after adjustment for multiple testing (Supplementary Table 5).

## Discussion

DNA-repair genes have a crucial role in the correction of damage to the genome of a cell and therefore their impairment can lead to carcinogenesis. Although these detrimental genetic alterations frequently originate within somatic cells during an individual's lifetime, a number of rare, hereditary mutations within specific DNA-repair genes have been identified that confer substantially increased risks to the individual of PrCa and other cancers. GWAS have also previously identified common, low-penetrance variants in close proximity to the DNA-repair genes *RAD51B* and *RAD23B* that contribute to PrCa susceptibility (Xu *et al*, 2012; Eeles *et al*, 2013; Amin Al Olama *et al*, 2015). However, even relatively well-powered genetic association studies may have been limited in their ability to reliably interrogate variants with lower MAFs or associations with modest effect sizes; therefore, additional-risk variants that confer their functional effect though DNA-repair genes may remain to be discovered. We have recently imputed PrCa data from the iCOGS study to the 1000 Genomes phase 3 reference panel, thereby enhancing the capability to interrogate this data set for untyped variants within tagged regions. In particular, a far greater number of lower MAF and insertion and deletion variants were available for analysis, although these are predominantly situated in non-coding regions. Imputation performance of lower MAF variants is improved by larger reference panel size and ethnic diversity and higher marker density on the genotyping array; however, rare variants still regularly remain challenging to impute without an additional reference panel enriched for specific low-frequency variants of known interest, and may also be more sensitive to differences in the imputation approach employed (Hoffmann and Witte, 2015). Our relatively large sample size provided good power to detect associations with PrCa for rare variants with greater effect sizes (e.g., for a variant at our 0.1% MAF cutoff with OR 2.5, we had 78% power) as well as common, low-penetrance variants (e.g., for a variant with OR 1.1 and a MAF of 20%, power was 86%). We were however limited with respect to the detection of variants with the combination of both modest allele frequency and effect size.

We have examined all variants in the imputed iCOGS data set situated within 20-kb of a panel of 179 DNA-repair genes for association with PrCa or more aggressive phenotypic presentation. No novel risk variants were identified in our single-SNP analysis, with only the previously reported signal at *RAD51B* on Chr14q24 genome-wide significant (Figure 1, Supplementary Table 3). Our analysis did not detect the previously reported signal at the *RAD23B* locus on Chr9q31, which was originally identified in the Chinese population and recently also confirmed in Europeans with the most significantly associated variant rs1771718 and the signal also an eQTL for *RAD23B* in normal prostate tissue in the TCGA data set (Xu *et al*, 2012; Amin Al Olama *et al*, 2015). rs1771718 is located ∼57 kb downstream of *RAD23B*, which is the closest neighbouring gene but located in a distinct recombination block from these risk variants. As no variant among the 509 within the gene centric region that we interrogated in this study showed substantial evidence for association (*P*⩾2.94 × 10^−3^), it appears likely that risk at this locus is modulated through a nearby regulatory element controlling expression of the gene as opposed to intragenic causal functional variants (Supplementary Figure 4).

We conducted two gene-level analyses in an attempt to identify whether there may be additional signals among the several loci that demonstrated suggestive but non-significant association peaks in our single-SNP analysis, but for which no individual variant had achieved significance. SKAT-C tests for the combined effects of common and rare variants, whereas SKAT-O adaptively combines the burden test and SKAT test in an attempt to maximise power for rare variant association testing (Lee *et al*, 2012; Ionita-Laza *et al*, 2013). We identified a significant PrCa-risk association after adjustment for multiple testing at the *MSH5* gene at Chr6p21 using the SKAT-C test, implying that multiple common, or a combination of common and rare variants within this gene may contribute to PrCa risk. Although caution must be taken with respect to this finding until replicated and deconstructed, this evidence implicates *MSH5* as a prospective PrCa susceptibility locus that warrants additional follow-up. *MSH5* had previously been reported as a plausible candidate gene for the lung cancer-risk locus at Chr6p21.33, for which the most strongly associated variant rs3117582 is intronic in *BAT3;* however, is highly correlated to rs3131379 in intron 10 of *MSH5* (Wang *et al*, 2008; Kazma *et al*, 2012). A recent study examining cancer pleiotropy among DNA-repair and DNA-damage signalling pathway variants has also reported a highly significant association with lung cancer for rs3115672, a synonymous variant within *MSH5*, in addition to weaker associations with colon and serous ovarian cancers (pleiotropic OR 1.18, 95% CI 1.12-1.24, *P*=2.53 × 10^−8^) (Scarbrough *et al*, 2016). This variant was however non-significant for PrCa within their study of 14 160 PrCa cases and 12 724 controls (OR 0.96, *P*=0.21). Within our larger study (of which 2614 cases and 2679 controls overlapped with those of Scarbrough *et al*), in the single-SNP analysis, rs3115672 remained non-significant after adjustment for multiple testing (OR 0.94, 95% CI 0.90–0.98, *P*=5.69 × 10^−3^). However, a number of other variants among the 312 within the *MSH5* gene in our analysis were more strongly associated, the top individual variant of which was rs9281573 (OR 0.94, *P*=4.01 × 10^−5^). StepAIC combined with SKAT leave one out selected two common and one rare variant as best explaining the SKAT-C association, all of which were among the top variants in the single-SNP analysis. This implies that a combination of common and rare variants could potentially underpin this signal.

We annotated these three variants for evidence of functionality using HaploReg v4.1 (Ward and Kellis, 2016); this annotation included chromatin state data for cell lines derived from multiple tissue types provided by the Roadmap Epigenomics Consortium (Roadmap Epigenomics *et al*, 2015); however, no data for prostate tissue were available. rs61036903, which is intronic to *MSH5*, showed limited direct evidence for functionality itself. Both of the variants situated around the *MSH5* promoter region, within *VWA7,* showed strong evidence for being located within enhancer elements that are active across a wide range of tissue types. In addition, expression data from the GTEx Consortium indicates that rs805825 is an eQTL for a number of genes from the MHC region (*HLA-DRB1*, *HLA-DRB5*, *LY6G5C*, *DDAH2*, *LY6G6C*, *HSPA1B* and *C4B*) (GTEx Consortium, 2015). These genes are clustered closely centromeric and telomeric of *MSH5* and *VWA7* within a gene dense locus; however, no eQTL with *MSH5* or *VWA7* was observed for this variant.

Although the *MSH5* gene is routinely classified as a member of the MMR pathway along with all other homologues of *MutS* (Wood *et al*, 2005; Ji *et al*, 2012; Scarbrough *et al*, 2016), functional evidence to date provides limited support for a role in MMR for *MSH5* itself. Instead, this gene has been implicated primarily in the processes of meiotic recombination, maintenance of chromosome integrity and DNA double-strand break repair (Clark *et al*, 2013; Wu *et al*, 2013). RNA-seq data from GTEx Analysis Release V6 for 2712 total samples across 51 normal human tissues (including 106 prostate tissue samples) demonstrates that *MSH5* is expressed at broadly similar levels across a wide range of tissue types, including prostate (GTEx Consortium, 2015; accessed via. http://www.gtexportal.org/home/gene/MSH5). Data from TCGA further support this expression profile across a range of normal tissues and also indicates that *MSH5* is consistently overexpressed for almost all tumour types in comparison with their respective normal tissues. For TCGA prostate tissue, a median RSEM (log2) value of 8.08 was observed across 498 tumour samples compared with 6.85 from 52 normal samples (http://cancergenome.nih.gov/; accessed via. http://firebrowse.org/viewGene.html?gene=msh5).

Taken together, these information demonstrate that although the *MSH5* gene represents a strong biological candidate for the PrCa-risk association that we have observed, additional functional follow-up studies will be required to dissect the precise functional variants, genes, regulatory elements or processes that underpin this signal.

It is worth noting that the gene level analyses in this study did not identify significant associations with any genes previously implicated in PrCa susceptibility. This was irrespective of whether the known risk mechanisms are believed to operate through multiple common, low-penetrance variants (e.g., *RAD51B*; SKAT-O *P*=0.05, SKAT-C *P*=2.76 × 10^−3^) or rare coding variants (e.g., *BRCA2*; SKAT-O *P*=0.46, SKAT-C *P*=0.15). In the case of *BRCA2* and other genes in which rare, moderate penetrance, protein truncating PrCa susceptibility variants had previously been identified, this is likely to reflect the fact that even using the latest 1000 Genomes reference panel, rare variants expected to confer greater phenotypic consequences may remain absent from the reference panel and consequently unimputable. This is consistent with the poor representation of coding insertion and deletion variants within our data set and would have rendered us underpowered to detect the effects of this class of variation in our analysis. Our observations do however imply that any additional contribution from common, lower penetrance variation at these genes may be minimal. This includes the rs11571833 nonsense polymorphism in the terminal exon of *BRCA2*, which is a reported lung cancer susceptibility variant, but was not associated with PrCa in this study (OR 1.03, 95% CI 0.89–1.19, *P*=0.74) (Wang *et al*, 2014). It is perhaps more surprising that *RAD51B* did not achieve significance under the SKAT-C test, which considers the potential contribution towards association of both common and rare variants within a region, given that three independent associations have previously been identified at this locus (Amin Al Olama *et al*, 2015). However, a suggestive association was observed under this test, which may be an indication that the cumulative effect size of the independent low-penetrance-risk variants within this region were insufficient to be conclusively disambiguated through this methodology.

Our pathway-level analysis identified suggestive but non-significant associations for two pathways under the SKAT-O test; translesion synthesis and mismatch repair. Although this study did not therefore provide sufficient evidence to implicate genes within these pathways in PrCa susceptibility, given the inherently conservative nature of the Bonferroni correction with respect to type II error and the relatively low proportion of coding variants within our data set, these observations may still justify further evaluation. In particular, as these suggestive associations were observed under the SKAT-O test that maximises power for rare variant association analyses and were not abrogated when the analyses were restricted only to coding variants, if substantiated, these nascent observations could be underpinned by direct effects of rare variants on the protein structure and function. Consequently, sequencing studies designed to comprehensively analyse the entire coding sequence of genes within the translesion synthesis and mismatch repair pathways could potentially yield further insight towards the mechanisms of susceptibility to developing PrCa. It is also worth noting that somatic mutations in translesion synthesis pathway genes, in particular the *POLK* gene, have been observed in prostate tumours previously (Makridakis and Reichardt, 2012; Yadav *et al*, 2015), whereas a rare germline nonsynonymous variant in the *POLI* gene has also been reported to predispose towards the occurrence of the TMPRSS2-ERG fusion in PrCa patients (Luedeke *et al*, 2009).

Increasing evidence suggests that moderate-penetrance germline mutations within DNA-repair genes also correlate with a more aggressive phenotypic presentation of PrCa and poorer prognosis (Castro *et al*, 2013; Cybulski *et al*, 2013; Leongamornlert *et al*, 2014; Robinson *et al*, 2015). This could in turn signify that DNA-repair gene variants might exist that do not confer greater risk of developing PrCa *per se*, yet do modify the likelihood of developing more aggressive disease in individuals that develop PrCa owing to other risk factors or exposures. We therefore also performed case–case analyses to further explore this hypothesis using two distinct phenotypic criteria. No significant or suggestive associations with aggressive disease were identified at the individual variant or gene levels under either definition; however, suggestive non-significant associations with the HR/FA pathway were observed. These analyses were, however, limited by relatively low sample numbers within each comparison group, which would have reduced our power to detect associations, particularly for rare and uncommon variants. We cannot therefore exclude the existence of additional DNA-repair gene variants that promote increased PrCa aggressiveness rather than risk of the disease itself; however, our data would suggest that any that exist are more likely to be rare than common.

Overall, this study represents the most comprehensive interrogation of the role of DNA-repair gene variants in PrCa susceptibility that we are aware of to date. We confirmed the presence of low-penetrance susceptibility loci situated at the *RAD51B* locus and found evidence to implicate a novel gene, *MSH5*, in PrCa susceptibility. We also share preliminary observations that rare germline variation in genes within the translesion synthesis pathway, in particular variants within the coding sequence, could be worthy of further investigation as candidates for PrCa risk.

The main limitations of our study relate to the challenges in imputing rare, potentially pathogenic variants to array genotype data from population-based reference panels and in performing association tests on low-frequency variants in a large multi-population study while controlling for population stratification. Therefore, additional sequencing studies would still be warranted to further explore the contribution of rare DNA-repair gene variants to PrCa risk. In addition, incomplete availability of phenotypic data and the fact that the iCOGS study did not specifically select individuals with low- or high-grade disease may have reduced our ability to examine any potential influence of these variants on PrCa aggressiveness. Future studies, whether array or sequencing based, that specifically select patients from these cohorts for inclusion would facilitate investigation of this aspect; which might in turn help to enhance stratification of patients that require altered clinical management pathways.

## Supplementary Material

Supplementary Figure 4

Supplementary Notes
