# Gene and pathway level analyses of germline DNA-repair gene variants and prostate cancer susceptibility using the iCOGS-genotyping array

**DOI:** 10.1038/bjc.2017.468

**Published:** 2018-02-13

**Authors:** Edward J Saunders, Tokhir Dadaev, Daniel A Leongamornlert, Ali Amin Al Olama, Sara Benlloch, Graham G Giles, Fredrik Wiklund, Henrik Grönberg, Christopher A Haiman, Johanna Schleutker, Børge G Nordestgaard, Ruth C Travis, David Neal, Nora Pasayan, Kay-Tee Khaw, Janet L Stanford, William J Blot, Stephen N Thibodeau, Christiane Maier, Adam S Kibel, Cezary Cybulski, Lisa Cannon-Albright, Hermann Brenner, Jong Y Park, Radka Kaneva, Jyotsna Batra, Manuel R Teixeira, Hardev Pandha, Koveela Govindasami, Ken Muir, The UK Genetic Prostate Cancer Study Collaborators, The UK ProtecT Study Collaborators, The PRACTICAL Consortium, Douglas F Easton, Rosalind A Eeles, Zsofia Kote-Jarai

**Correction to:** *British Journal of Cancer* (2016) **114**, 945–952; doi:10.1038/bjc.2016.50; published online 10 March 2016

Following the publication of this manuscript, it was recognised that some of the names included in the list of The UK Genetic Prostate Cancer Study Collaborators had not been presented in full. Full names have now been provided for the majority of these collaborators and their names appear correctly below:

Mohammed L Al-Sudani, Dr Mark Beresford, Barnaby G Chappell, Marcus J Drake, Dr Baba M Ghana, Donald Macdonald, Shoukat H Memon, Moiketse Mokete, Jan D Nawrocki, Dr Graham Read, Edward W J Rowe, Dr Richard E Shaffer, Dr Lucy Side, Vaikantum Srinivasan.

