# Supplementary figures and images for: Gene and pathway level analyses of germline DNA-repair gene variants and prostate cancer susceptibility using the iCOGS-genotyping array
Source: Br J Cancer. 2016 Mar 10;114(8):945–52. doi: 10.1038/bjc.2016.50 (PMC5379914; doi:10.1038/bjc.2016.50)

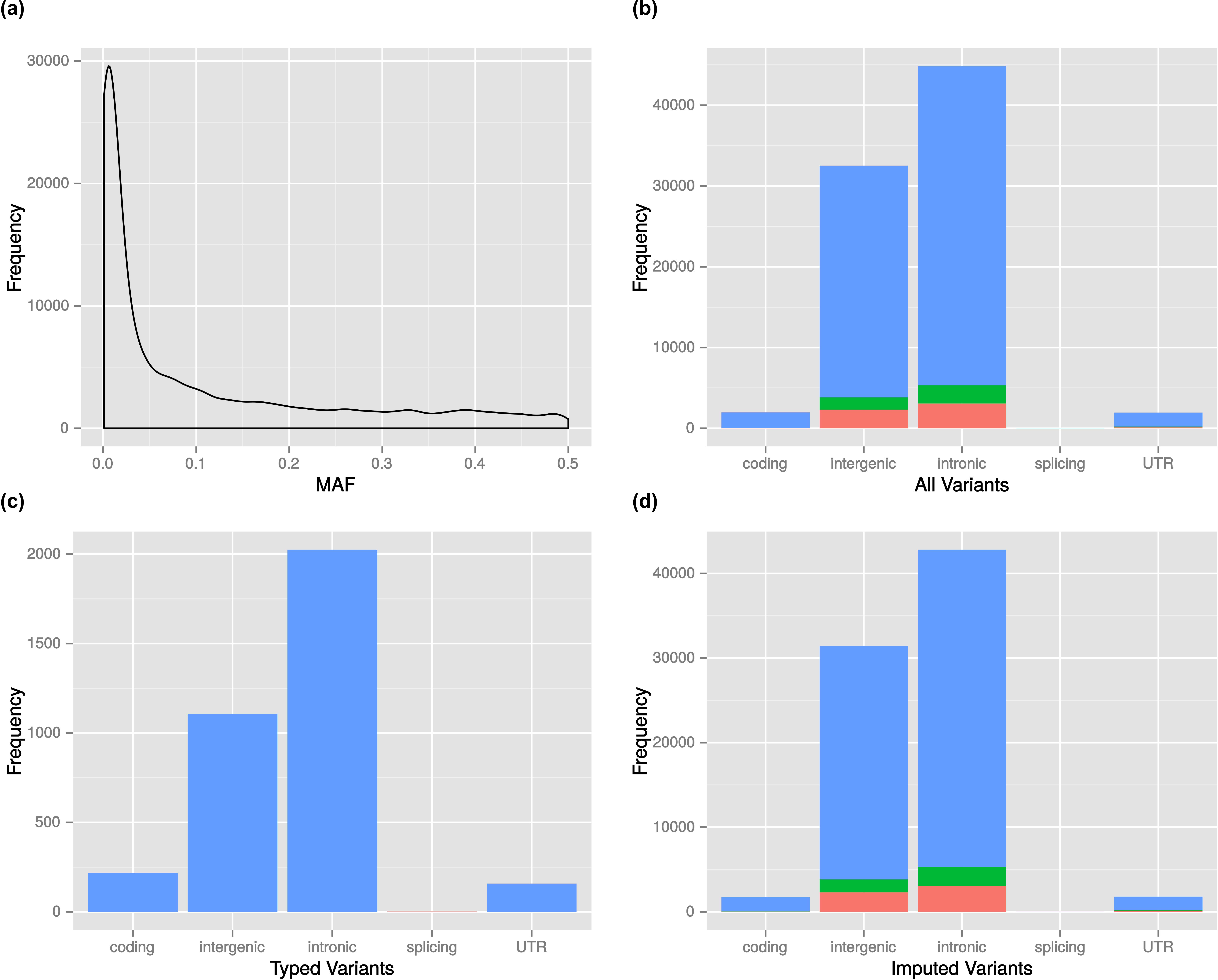

Supplement: Supplementary Figure 1 [file bjc201650x1.tif]

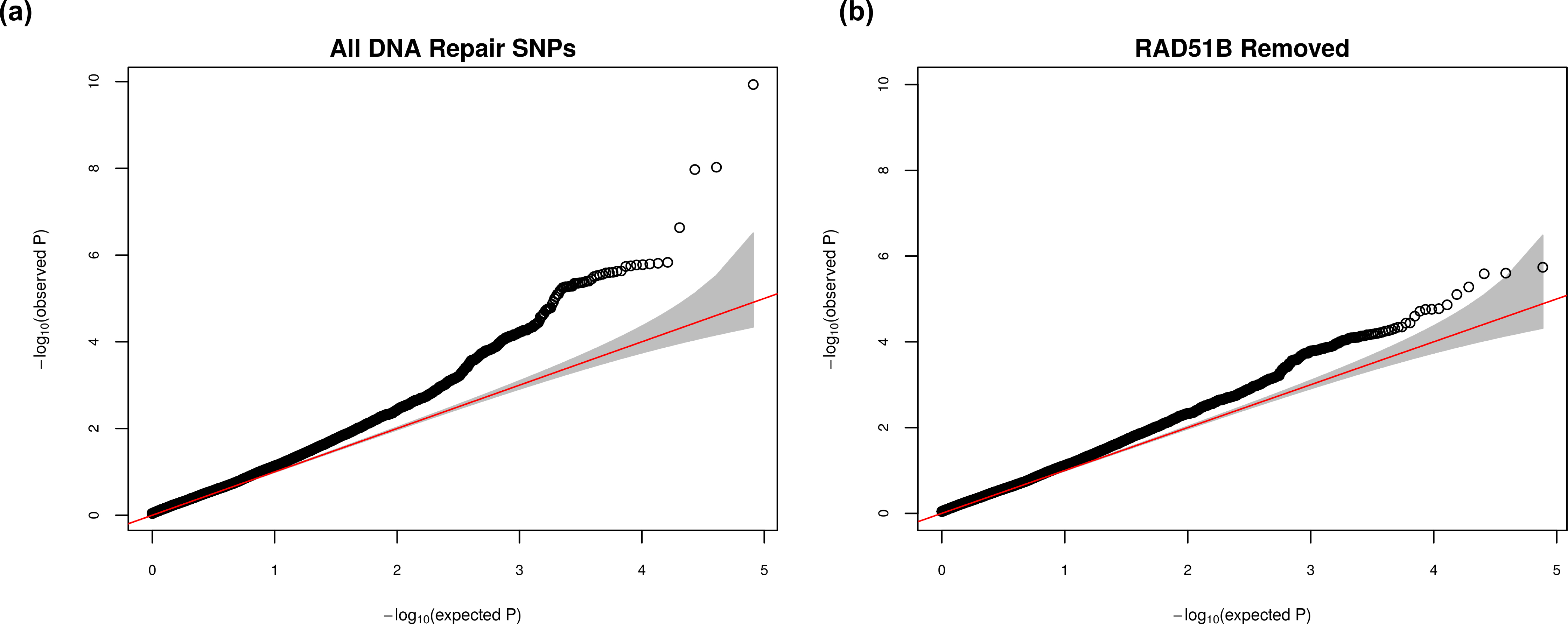

Supplement: Supplementary Figure 2 [file bjc201650x2.tif]

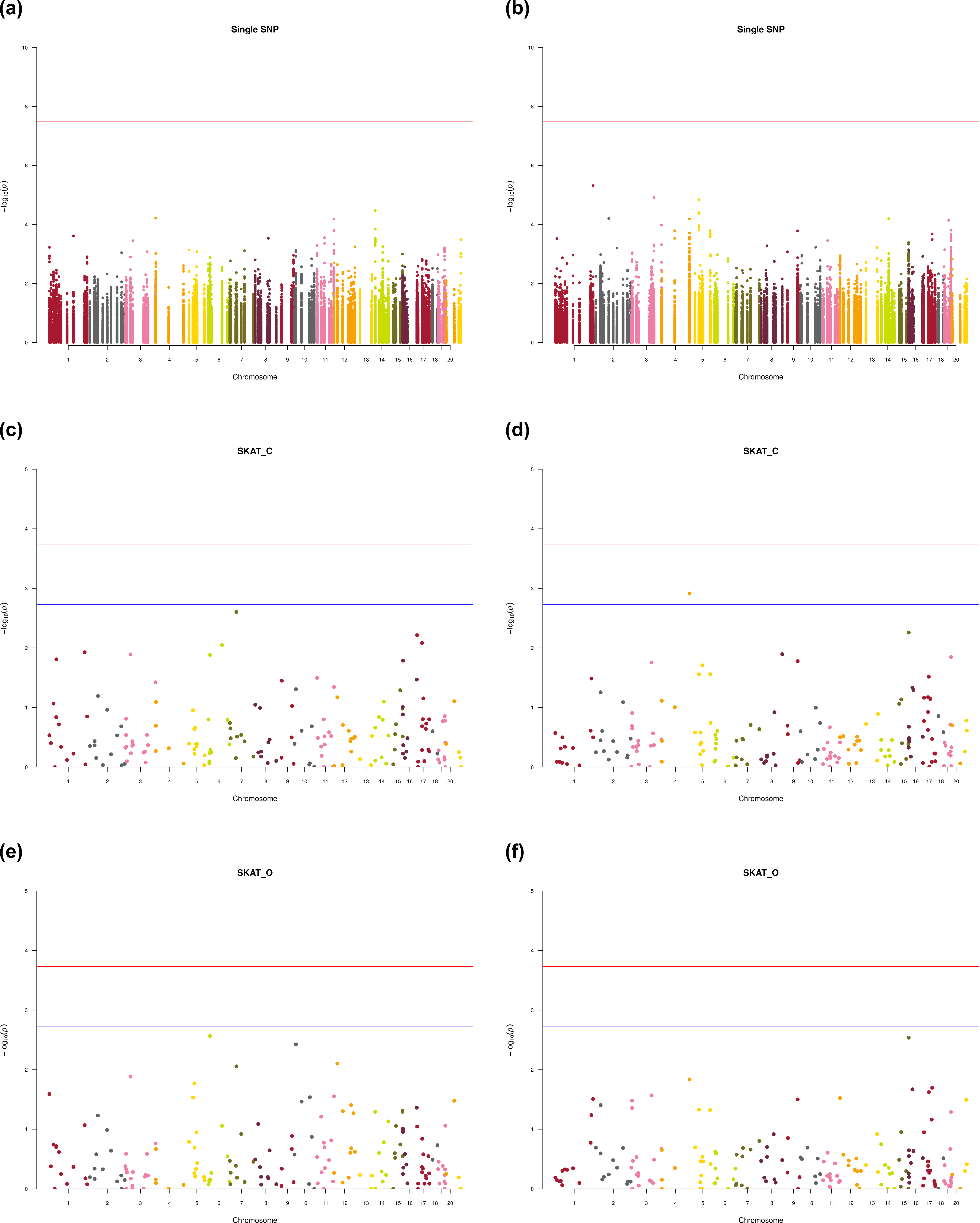

Supplement: Supplementary Figure 3 [file bjc201650x3.tif]

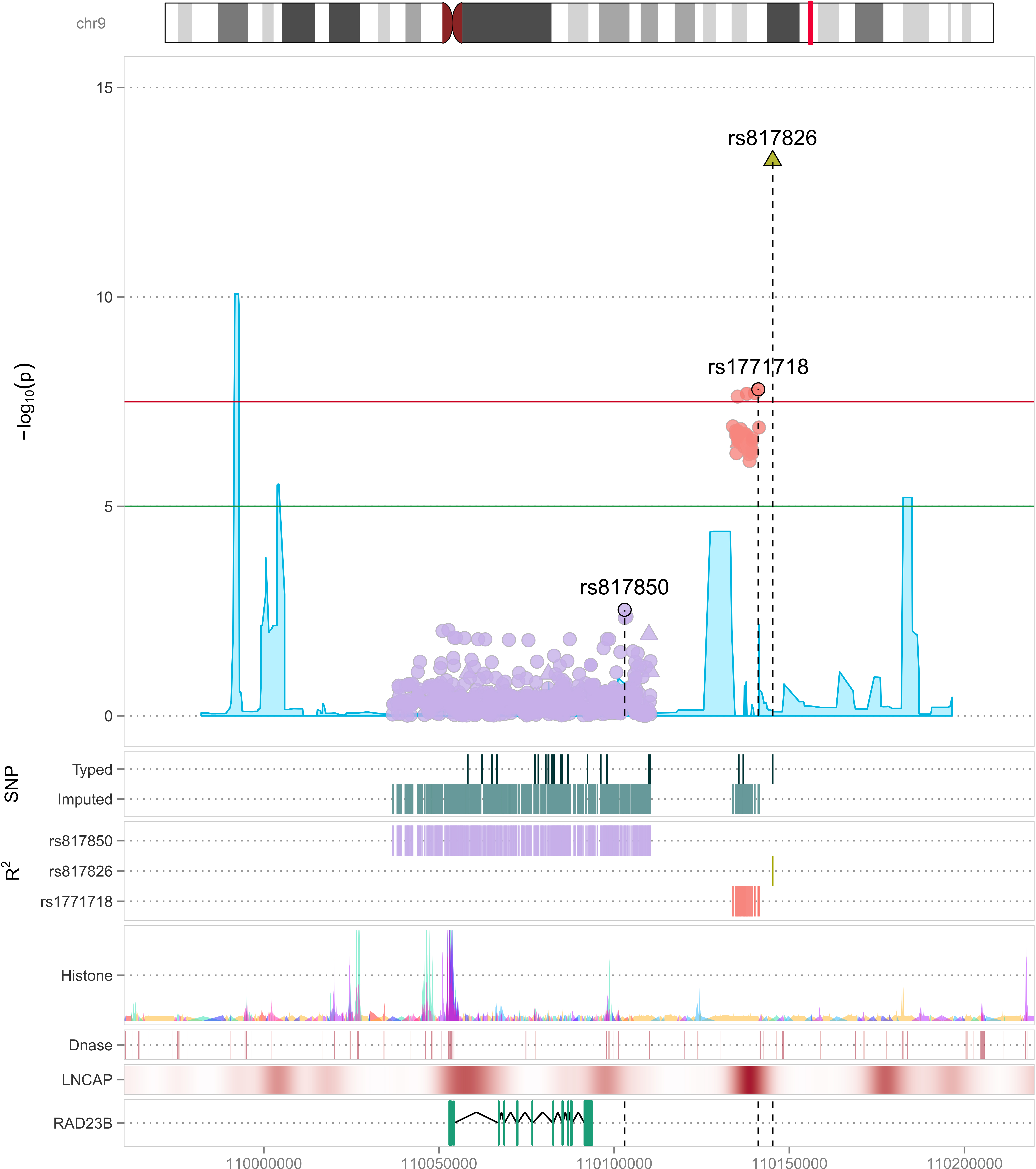

Supplement: Supplementary Figure 4 [file bjc201650x4.tif]
