# Supplementary material for: Gene and pathway level analyses of germline DNA-repair gene variants and prostate cancer susceptibility using the iCOGS-genotyping array
Source: Br J Cancer. 2016 Mar 10;114(8):945–52. doi: 10.1038/bjc.2016.50 (PMC5379914; doi:10.1038/bjc.2016.50)
Supplement: Supplementary Notes [file bjc201650x5.docx]

**Supplementary Note:**

**The PRACTICAL CONSORTIUM (in addition to those named in the author list)**

Information on the consortium can be found at <http://practical.ccge.medschl.cam.ac.uk/>

Additional members from the consortium are: Margaret Cook ^1^, Angela Morgan ^2^, Artitaya Lophatananon ^3,4^, Cyril Fisher ^2^, Malgorzata Tymrakiewicz ^2^, Michelle Guy ^2^, Rosemary Wilkinson ^2^, Sara Jugurnauth-Little ^2^, Steve Hazel ^2^, Melissa C. Southey ^5^, Liesel M. Fitzgerald ^6^, John Pedersen ^7^, John Hopper ^8^, Ami Karlsson ^9^, Carin Cavalli-Bjoerkman ^9^, Jan-Erik Johansson ^9^, Jan Adolfson ^9^, Markus Aly ^9,10^, Michael Broms ^9^, Paer Stattin ^9^, Brian E. Henderson ^11^, Fredrick Schumacher ^11^, Anssi Auvinen ^12^, Kimmo Taari ^13^, Liisa Maeaettaenen ^14^, Paula Kujala ^15^, Teemu Murtola ^16,17^, Teuvo LJ Tammela ^17^, Tiina Wahlfors ^18^, Andreas Roder ^19^, Peter Iversen ^19^, Peter Klarskov ^20^, Sune F. Nielsen ^21,22^, Tim J. Key ^23^, Hans Wallinder ^24^, Sven Gustafsson ^24^, Jenny L. Donovan ^25^, Freddie Hamdy ^26^, Angela Cox ^27^, Anne George ^28^, Athene Lane ^28^, Gemma Marsden ^26^, Michael Davis ^25^, Paul Brown ^25^, Paul Pharoah ^29^, Lisa B. Signorello ^31,30^, Wei Zheng ^32^, Shannon K. McDonnell ^33^, Daniel J. Schaid ^33^, Liang Wang ^33^, Lori Tillmans ^33^, Shaun Riska ^33^, Thomas Schnoeller ^34^, Kathleen Herkommer ^35^, Manuel Luedeke ^34^, Walther Vogel ^36^, Dominika Wokolorczyk ^37^, Jan Lubiski ^37^, Wojciech Kluzniak ^37^, Katja Butterbach^38^, Christa Stegmaier ^39^, Bernd Holleczek^39^, Hui-Yi Lin^40^, Hyun Park ^41^, Julio Pow-Sang ^41^, Thomas Sellers ^41^, Chavdar Slavov ^42^, Aleksandrina Vlahova ^43^, Atanaska Mitkova ^44^, Darina Kachakova ^44^, Elenko Popov ^42^, Svetlana Christova ^43^, Tihomir Dikov ^43^, Vanio Mitev ^44^, Allison Eckert ^45^, Amanda Spurdle ^46^, Angus Collins ^45^, Glenn Wood ^45^, Greg Malone ^45^, Judith A. Clements ^45^, Kris Kerr ^45^, Megan Turner ^45^, Pamela Saunders ^45^, Peter Heathcote ^45^, Srilakshmi Srinivasan ^45^, Leire Moya ^45^, Trina Yeadon ^45^, Australian Prostate Cancer BioResource ^45^, Joana Santos ^47^, Carmen Jerónimo^47^, Paula Paulo ^47^, Pedro Pinto ^47^, Rui Henrique ^47^, Sofia Maia ^47^, Agnieszka Michael ^48^, Andrzej Kierzek ^48^, Huihai Wu ^48^

^1^Centre for Cancer Genetic Epidemiology, Department of Public Health and Primary Care, University of Cambridge, Strangeways Laboratory, Worts Causeway, Cambridge CB1 8RN, UK, ^2^The Institute of Cancer Research, Sutton, UK, ^3^Institute of Population Health, University of Manchester, Manchester, UK, ^4^Warwick Medical School, University of Warwick, Coventry, UK, ^5^ Genetic Epidemiology Laboratory, Department of Pathology, The University of Melbourne, Grattan Street, Parkville, Victoria 3010, Australia, ^6^ Cancer Epidemiology Centre, The Cancer Council Victoria, 615 St Kilda Road, Melbourne, Victoria, Australia, ^7^ Tissupath Pty Ltd., Melbourne, Victoria 3122, Australia, ^8^ Centre for Epidemiology and Biostatistics, Melbourne School of Population and Global Health, The University of Melbourne, Victoria, Australia, ^9^Department of Medical Epidemiology and Biostatistics, Karolinska Institute, Stockholm, Sweden, ^10^Department of Clinical Sciences at Danderyds Hospital, Stockholm, Sweden, ^11^Department of Preventive Medicine, Keck School of Medicine, University of Southern California/Norris Comprehensive Cancer Center, Los Angeles, California, USA, ^12^Department of Epidemiology, School of Health Sciences, University of Tampere, Tampere, Finland, ^13^Department of Urology, Helsinki University Central Hospital and University of Helsinki, Helsinki, Finland, ^14^Finnish Cancer Registry, Helsinki, Finland, ^15^ Fimlab Laboratories, Tampere University Hospital, Tampere, Finland, ^16^School of Medicine, University of Tampere, Tampere, Finland, ^17^Department of Urology, Tampere University Hospital and Medical School, University of Tampere, Finland, ^18^BioMediTech, University of Tampere and FimLab Laboratories, Tampere, Finland, ^19^Copenhagen Prostate Cancer Center, Department of Urology, Rigshospitalet, Copenhagen University Hospital, Tagensvej 20, 7521, DK-2200 Copenhagen, Denmark, ^20^Department of Urology, Herlev Hospital, Copenhagen University Hospital, Herlev Ringvej 75, DK-230 Herlev, Denmark, ^21^Department of Clinical Biochemistry, Herlev Hospital, Copenhagen University Hospital, Herlev Ringvej 75, DK-230 Herlev, Denmark, ^22^Faculty of Health and Medical Sciences, University of Copenhagen, ^23^Cancer Epidemiology Unit, Nuffield Department of Clinical Medicine, University of Oxford, Oxford, UK, ^24^ Department of Epidemiology and Biostatistics, School of Public Health, Imperial College, London, UK, ^25^ School of Social and Community Medicine, University of Bristol, Canynge Hall, 39 Whatley Road, Bristol, BS8 2PS, UK, ^26^ Nuffield Department of Surgical Sciences, University of Oxford, Oxford, UK, Faculty of Medical Science, University of Oxford, John Radcliffe Hospital, Oxford, UK, ^27^ CR-UK/YCR Sheffield Cancer Research Centre, University of Sheffield, Sheffield, UK, ^28^ University of Cambridge, Department of Oncology, Box 279, Addenbrooke's Hospital, Hills Road Cambridge CB2 0QQ, UK, ^29^ Centre for Cancer Genetic Epidemiology, Department of Oncology, University of Cambridge, Strangeways Laboratory, Worts Causeway, Cambridge, UK, ^30^ International Epidemiology Institute, 1555 Research Blvd., Suite 550, Rockville, MD 20850, USA, ^31^ Department of Epidemiology, Harvard School of Public Health, 677 Huntington Avenue, Boston, MA 02115, USA, ^32^ Division of Epidemiology, Department of Medicine, Vanderbilt University Medical Center, 2525 West End Avenue, Suite 800, Nashville, TN 37232 USA, ^33^ Mayo Clinic, Rochester, Minnesota, USA, ^34^ Department of Urology, University Hospital Ulm, Germany, ^35^ Department of Urology, Klinikum rechts der Isar der Technischen Universitaet Muenchen, Munich, Germany, ^36^ Institute of Human Genetics, University Hospital Ulm, Germany, ^37^ International Hereditary Cancer Center, Department of Genetics and Pathology, Pomeranian Medical University, Szczecin, Poland, ^38^Division of Clinical Epidemiology and Aging Research, German Cancer Research Center (DKFZ), 69120 Heidelberg, Germany, ^39^Saarland Cancer Registry, 66119 Saarbruecken, Germany, ^40^Biostatistics Program, Moffitt Cancer Center, 12902 Magnolia Drive, Tampa, FL 33612, USA, ^41^Department of Cancer Epidemiology, Moffitt Cancer Center, 12902 Magnolia Drive, Tampa, FL 33612, USA, ^42^Department of Urology and Alexandrovska University Hospital, Medical University, Sofia, Bulgaria, ^43^Department of General and Clinical Pathology, Medical University, Sofia, Bulgaria, ^44^Department of Medical Chemistry and Biochemistry, Molecular Medicine Center, Medical University, Sofia, 2 Zdrave Str., 1431 Sofia, Bulgaria, ^45^Australian Prostate Cancer Research Centre-Qld, Institute of Health and Biomedical Innovation and School of Biomedical Science, Queensland University of Technology, Brisbane, Australia, ^46^Molecular Cancer Epidemiology Laboratory, Queensland Institute of Medical Research, Brisbane, Australia, ^47^Department of Genetics, Portuguese Oncology Institute, Porto, Portugal, ^48^The University of Surrey, Guildford, Surrey, GU2 7XH, UK

**The UK Genetic Prostate Cancer Study Collaborators**

Information on the UKGPCS study can be found at <http://www.icr.ac.uk/our-research/research-divisions/division-of-genetics-and-epidemiology/oncogenetics/research-projects/ukgpcs>

Mr Z Abbasi, Mr M Akhlil Abdul-Hamid, Mr Paul D Abel, Professor Paul H Abrams, Dr Fawzi A Adab, Mr Andrew Adamson, Mr A Adeyoju, Mr Naveed Afzal, Mr Ernest K N Ahiaku, Mr Munir Ahmed, Mr Mohammed L Al Sudani, Dr Christopher Alcock, Dr Zulfiqar Ali, Mr David J Almond, Dr Roberto Alonzi, Dr Amir S M Al-Samarraie, Dr Al-Samerraie, Mr Waleed Al-Singary, Mr Al-Sudani, Mr John Anderson, Mr Steven Andrews, Mr Henry Andrews, Mr Iqbal Anjum, Mr Ken Anson, Dr Nicola A Anyamene, Mr Ike Apakama, Dr F Aparcia, Mr J A A Archbold, Dr D Ash, Dr Richard F U Ashford, Dr A Azzabi, Mr David Badenoch, Dr Amit Bahl, Mr M J Bailey, Mrs Karen Bailey, Mr Andrew J Ball, Mr G Banerjee, Dr N Barber, Dr Jim Barber, Dr Baria, Mr Douglas G Barnes, Mr J Bashir, Mr Pradip Basu, Mr Christopher A Bates, Dr N A Bax, Mr D Baxter-Smith, Mr Amar Bdesha, Mr Christopher J M Beacock, Professor Ronald P Beaney, Mr Ralph Beard, Mr John D Beatty, Mr Rupert Beck, Ms Gail Beese, Dr Sharon Beesley, Mr C Richard W Bell, Mr James Bellringer, Dr Richard Benson, Dr Beresford, Mr Christopher R A Bevis, Dr Rajanee Bhana, Mr S Bhanot, Dr A Bhatnagar, Mr R I Bhatt, Mr Brian Birch, Dr Alison Birtle, Mr M Bishop, Mr C Shekhar Biyani, Mr A R E Blacklock, Miss Rosemary Blades, Dr Peter Bliss, Dr David J Bloomfield, Miss S Boddy, Professor C M Booth, Mr Pradeep Bose, Dr Michael C Bott, Dr David Bottomley, Mr Nigel R Boucher, Dr J Bowen, Dr Mark Bower, Mr W G Bowsher, Mr P J R Boyd, Mr F James Bramble, Mr Simon F Brewster, Mr Tim Briggs, Dr Cathryn Brock, Dr Sue Brock , Mr Stephen Bromage, Mr Richard Brough, Dr Richard Brown, Mr Stephen Brown, Mr Richard Brown, Mr Tony J Browning, Mr N Bryan, Mr Neil A Burgess, Mr Nicholas Burns-Cox, Mr Paul C Butterworth, Mr D Cahill, Mr P S Callaghan, Mr John Calleary, Dr M Calleja, Dr Frances Calman, Dr Philip Camilleri, Mr Alister Campbell, Miss Andrea Cannon, Dr Dawn M Carnell, Mr T W Carr, Mr Simon Carter, Mr Charles J M Carter, Dr Adam C Carter, Dr Bruce M Castle, Mr David Chadwick, Mr Rohit Chahal, Dr P Chakraborti, Mr Chappell, Mr C Charig, Dr Anula D Chetiyawardana, Mr Christopher Chilton, Mr F I Chinegwundoh, Dr Irene Chong, Dr Ananya Choudhury, Mr Wai-Man Chow, Mr Timothy J Christmas, Dr Mark J Churn, Mr Noel W Clarke, Mr Jorge Clavijo-Eisele, Dr M Coe, Mr N P Cohen, Mr C Coker, Dr Trevor Cole, Dr David J Cole, Mr O Cole, Mr Gerald Collins, Dr Matthew Collinson, Mr I Conn, Dr C Connell, Dr Audrey Cook, Mr Peter Cooke, Mr Graeme Cooksey, Mr L Coombs, Mr Robert F Copland, Mr Andrew J Cornaby, Mr P A Cornford, Mr Corolis, Mr John Corr, Mr C B Costello, Mrs N Coull, Dr Richard Cowan, Mr Robert Cox, Dr C Coyle, Mr Jeremy Crew, Mr John C Crisp, Dr W Cross, Mr W Cross, Dr Dorthe Cruger, Mr Malcolm Crundwell, Mr Cummings, Mr Nazeer Dahar, Dr Francis N Daniel, Mr J Darrad, Mr Pallon Daruwala, Mr Gautam Das, Mr Shibendra Datta, Dr S Davidson, Dr Joseph Davies, Mr Owen W Davison, Mr Guy Dawkins, Mr Chris Dawson, Mr Alan R De Bolla, Professor David Dearnaley, Mr Ken M Desai, Dr George P Deutsch, Mr John Dick, Mr Andrew J Dickinson, Dr Jeanette Dickson, Mr Michael Dinneen, Dr Sanjay Dixit, Dr H Jane Dobbs, Mr A Doble, Dr David Dodds, Mr Alan Doherty, Mr P Donaldson, Dr M Dooldeniya, Dr S Fiona Douglas, Mr Drake, Dr Gill M Duchesne, Mr Peter Duffy, Mr Michael Dunn, Mr W D Dunsmuir, Dr Sajid K Durrani, Mr Alan C Eaton, Professor Diane Eccles, Mr B Eddy, Mr C D Eden, Mr J Edwards, Mr Jeremy Elkabir, Dr P Tony Elliott, Mr B W Ellis, Dr R Ellis, Dr A El-Modir, Mr Andrew W S Elves, Dr Christine Elwell, Mr Mark Emberton, Dr Louise Emmerson, Mr Roland C D England, Mr R D Errington, Professor D Gareth Evans, Dr Alison Falconer, Mr Derek Fawcett, Dr C Featherston, Dr Carolyn J Featherstone, Mr Jeremy Feggetter, Dr C Ferguson, Dr D Fermont, Mr Michael Ferro, Mr Matthew Fletcher, Dr A Folkes, Mr Trevor F Ford, Mr Paul W Foster, Dr Kevin N Franks, Dr Olivera Frim, Dr Joanna Gale, Mr Christopher Gallegos, Mr James S Gelister, Dr Ghana, Dr Stephanie Gibbs, Mr Hugh Gilbert, Mr David Gillatt, Dr John Glaholm, Mr Jonathan M Glass, Mr James Glenister, Dr Thomas D Goode, Ms E M Gordon, Mr Richard L Gower, Dr John Graham, Mr Damian Green, Mr Jonathan Greenland, Dr Robert Grieve, Mr Thomas R L Griffiths, Mr Sandy Gujral, Dr Nishi Gupta, Mr Riza Murat Gurun, Mr Peter J Guy, Mr Neil Haldar, Mr N Halder, Professor F C Hamdy, Dr C Hamilton, Mr John Hammonds, Mr S J Hampson, Mr Damien C Hanbury, Dr P D John Hardman, Dr Stephen J Harland, Mr John M Harney, Dr Peter Harper, Dr Sarah Harris, Mr D Harris, Mr G S M Harrison, Mr D R Harriss, Mr N Harvey-Hills, Mr Simon Hawkyard, Dr Catherine M Heath, Mr Michael Hehir, Mr Giles O Hellawell, Mr David Hendry, Mr Mike Henley, Dr Ann Henry, Dr John Hetherington, Dr Tamas Hickish, Mr James A Hicks, Dr Serena Hilman, Mr Richard Hindley, Mr John R Hindmarsh, Mr John Hines, Dr M Hingorani, Mr Edwin T S Ho, Professor Shirley Hodgson, Dr U Hoffman, Mr David Holden, Dr A Hollingdale, Mr Graham W Hollins, Mr Simon A V Holmes, Dr Gail Horan, Professor Alan Horwich, Professor Peter Hoskin, Mr Graham P Howell, Mr D Hrouda, Dr Robert Huddart, Ms Liz Hudson, Dr Rob Hughes, Mr Michael Hughes, Mr Owen Hughes, Dr Caroline Humber, Mr John W Iacovou, Dr A Ibrahim, Mr John A Inglis, Mr Stuart Irving, Mr C Irwin, Dr Louise Izatt, Mr Victor Izegbu, Mr Basharat Jameel, Mr Michael J James, Professor N James, Mr R Lester James, Mr Pradip Javle, Dr P Jenkins, Dr Sameer Jhavar, Dr Gareth Jones, Mr Chris R Jones, Dr David A Jones, Mr J Joseph, Dr Shelagh Joss, Mr Amir Kaisary, Dr Alexandre L Kaliski, Dr G Kapur, Mr O Karim, Dr Stephen J Karp, Mr F X Keeley, Mr Anand R Kelkar, Mr J P Kelleher, Mr John Kelly, Dr Sue Kenwrick, Mr F Khan, Dr Vincent Khoo, Ms Rachel M Kimber, Mr R Kinder, Professor Roger S Kirby, Professor David Kirk, Dr Peter Kirkbride, Mr Magdi M Kirollos, Mr Roger Kockelbergh, Mr Philip C W C Koenig, Mr Gordon G Kooiman, Dr O Koreich, Mr Anthony Koupparis, Mr Mohamed Kourah, Dr Sigurd Kraus, Ms Magda L Kujawa, Mr Ravi Kulkarni, Mr M Kumar, Dr Ian H Kunkler, Professor H Kynaston, Dr Katherine L Lachlan, Dr Robert Laing, Dr Fiona Lalloo, Mr M Lancashire, Mr Stephen E M Langley, Mr Marc Laniado, Mr T R Larner, Mr Maurice W Lau, Mr W T Lawrence, Miss Anne Lawson, Mr Pieter J Le Roux, Professor Mary Leader, Mr J O Lee, Ms L Lee, Ms A Lee, Dr R John Lemburger, Dr Priscilla Leone, Dr Jason Lester, Mr Hing Leung, Mr J Lewis, Mr D Christopher Lewis, Mr Thomas Liston, Dr Jacqueline Livsey, Mr S Lloyd, Dr Imogen Locke, Mr Richard Lodge, Dr John Logue, Mr Mark Longmuir, Mr Malcolm G Lucas, Mr C J Luscombe, Dr Anna Lydon, Mr Michael Lynch, Mr Naing N K Lynn, Mr James P A MacDermott, Mr Ruaraidh P Macdonagh, Mr Macdonald, Mr Sanjeev Madaan, Dr Kudingila R Madhava, Dr Joseph Maguire, Professor E R Maher, Dr Rana Mahmood, Dr Graeme H M Mair, Mr Peter R Malone, Dr Stephen A Mangar, Mr Mark Mantle, Mr I Mark, Mr Robert Mason, Professor M D Mason, Mr Matanhelia, Mr Shyam Matenhelia, Mr Philip N Matthews, Dr J McAleese, Ms Donna McBride, Mr Jonathan McFarlane, Mr McGrath, Mr Craig McIlhenny, Mr Paul McInerney, Mr Gregor McIntosh, Dr F McKinna, Dr Duncan McLaren, Miss Esther McLarty, Dr Rhona McMenemin, Mr Alan McNeill, Mr T A McNicholas, Mr Robert N Meddings, Mr A David Mee, Dr Lucinda Melcher, Mr Memon, Mr Pravin Menzes, Mr Marek Miller, Mr Robert Mills, Mr S Mitchell, Dr Natasha Mithal, Dr Anita Mitra, Ms Gillian E Mobb, Mr Leslie E F Moffat, Mr Mokete, Dr Julian Money-Kyrle, Mr Bruce Montgomery, Mr Martin P Moody, Mr Roland Morley, Mr Sean B Morris, Professor Patrick Morrison, Dr Diana Mort, Mr Amir H Mostafid, Mr Hanif Motiwala, Mr Gulzar Mufti, Mr Gordon Muir, Mr Faiz Mumtaz, Mr Michael Murphy, Mr Keith W Murray, Dr Alexandra Murray, Dr Shirley Murrell, Dr D Muthukumar, Mr Harry Naerger, Mr Siva Namasivayam, Mr Vinod Nargund, Mr Nawrocki, Mr Donald Neilson, Dr A Nethersell, Mr Julian Barwell, Dr Jacqueline C Newby, Dr Hugh Newman, Dr R Newton, Mr Neil Oakley, Mr P J O'Boyle, Mr J O'Brien, Mr Tim S O'Brien, Dr H O'Donnell, Mr Neil O'Donoghue, Mr E O'Donoghue, Mr Chris Ogden, Mr Hemant Ohja, Professor Tim Oliver, Mr Eng K Ong, Mr P O'Reilly, Dr J S O'Rourke, Mr David Osborn, Dr Peter Ostler, Professor Joe O'Sullivan, Dr J Owen, Mr Edward Palfrey, Dr Miguel Panades, Dr Niki Panakis, Mr M Pancharatnam, Mr Michalakis L Pantelides, Dr U Panwar, Dr Omi Parikh, Dr Chris Parker, Mr Christopher H Parker, Mr Bohdan T Parys, Dr Sarah Pascoe, Mr Anup Patel, Dr Joan Paterson, Mr S Pathack, Ms Jhumur Pati, Dr Helen Patterson, Dr Pattu, Mr A Paul, Dr Heather Payne, Dr David Peake, Dr I Pedley, Mr A Pengelly, Mr Amjad M Peracha, Dr Matthew Perry, Mr Raj Persad, Mr John Peters, Mr N H Philp, Mr T Philp, Dr Lisa M Pickering, Dr Katharine Pigott, Mr R Plail, Dr P Nicholas Plowman, Mr Richard D Pocock, Mr A J Pope, Mr Rick Popert, Mr Tim Porter, Mr John M Potter, Mr Christopher Powell, Dr Thomas B Powles, Mr Krishna Prasad, Mr Seshadri Sri Prasad, Mr J W Prejbisz, Mr Stephen Prescott, Dr Andrew Protheroe, Mr Khaver N Qureshi, Dr Nigel Raby, Dr Narasimhan Ragavan, Mr Palaniappa G S Raju, Dr Prakash B Ramachandra, Dr R Raman, Mr Abhay Rane, Dr Julia Rankin, Mr Y Rao, Mr Hari L Ratan, Mr Ramachandran Ravi, Dr K Ravishankar, Dr Read, Mr Paul J Reddy, Mr Peter R Rimington, Dr Peter A Ritchie, Dr J Trevor Roberts, Mr Andrew Robertson, Dr Angus Robinson, Dr Anne C Robinson, Mr Lee Q Robinson, Mr Mark A Rochester, Mr P B Rogers, Mr Tomas P Rosenbaum, Mr Neil Rothwell, Mr Carl Rowbotham, Mr Rowe, Dr Kathryn Rowley, Dr Deborah Ruddy, Mr John Rundle, Dr John M Russell, Mr P G Ryan, Dr A Sabharwal, Dr Anand K Saggar, Dr Ali Samanci, Mr Vijay K Sangar, Mr M F Saxby, Mr Hartwig Schwaibold, Dr John E Scoble, Dr Christopher Scrase, Mr Selim, Mr Henry Sells, Mr Krishna K Sethia, Mr David C Shackley, Dr Shaffer, Dr Nihil Shah, Dr D Shakespeare, Dr Sue Shanley, Mr Neerah K Sharma, Dr Denise J Sheehan, Dr Elizabeth Sherwin, Dr Poh Lin Shum, Dr LucySide, Dr Norma Sidek, Professor Karol Sikora, Dr R Simcock, Mr Andrew M Sinclair, Mr Pravin Singh, Dr M Siva, Mr Michael F Smith, Mr James Smith, Dr Michael Sokal, Mr Graham M Sole, Mr Mark J Speakman, Dr Alexander Spiers, Dr Thiagarajan Sreenivasan, Dr Narayanan N Srihari, Mr Srinivasan, Mr Rajagopalan Sriram, Dr John N Staffurth, Dr D Stewart, Dr Andrew Stockdale, Mr Mark A Stott, Mr M J Stower, Mr John R Strachan, Professor Nicholas S A Stuart, Dr Elaine Sugden, Mr Duncan Summerton, Dr Santhanam Sundar, Mr S K Sundaram, Mr Gokarakonda Suresh, Mr Shabbir Susnerwala, Mr Kuchibhotla S Swami, Miss Stephanie J Symons, Dr Isabel Syndikus, Dr Saad Tahir, Dr J Tanquay, Dr John W Taylor, Dr J W Taylor, Mr T Terry, Dr Robert J Thomas, Mr Stephen A Thomas, Mr Alan Thompson, Dr Alastair H Thomson, Dr A Thurston, Dr Owen Tilsley, Mr Stuart F Tindall, Dr K Tipples, Dr Tong, Mr Hamid Toussi, Dr Elizabeth W Toy, Professor Richard C Trembath, Mr David N Tulloch, Mr Kevin J Turner, Mr James Tweedle, Dr C J Tyrell, Mr N Umez-Eronini, Mr Graeme H Urwin, Mr Justin A Vale, Dr Van As, Dr Nicholas Van As, Dr Subramaniam Vasanthan, Mr Sean Vesey, Dr Maria Vilarino-Varela, Dr John Violet, Mr Jaspal Virdi, Dr Robert Wade, Dr Katherine Waite, Mr E M Walker, Mr Roger Walker, Mr David M A Wallace, Mr Nicholas A Watkin, Mr M E Watson, Professor J H Waxman, Mr Brian Waymont, Dr Andrew Weaver, Mr Ralph J Webb, Mr Andrew Wedderburn, Dr Paula Wells, Mr G D Wemyss-Holden, Mr P M T Weston, Dr Duncan Wheatley, Mr P Whelan, Dr D Whillis, Mr Adam D Wilde, Dr Vicki Wiles, Dr Marie Wilkins, Mr John H Williams, Mr Simon Williams, Mr Michael Willis, Mr Michael I Wills, Mr Richard Wilson, Mr J R Wilson, Mr Mathias H Winkler, Dr Marcus Wise, Mr Simon Woodhams, Professor C Woodhouse, Dr Cathryn Woodward, Dr Woolf, Mr K A Woolfenden, Dr Jane Worlding, Mr Mark Wright, Dr WYLIE, Dr James P Wylie, Dr Chris Wynne, Ms Angelika Zang, Dr A Zarkar,

**The UK ProtecT Study Collaborators**

Angela Cox**,** Paul M. Brown**,** Anne George**,** Gemma Marsden**,** Athene Lane**,** Michael DavisPrasad Bollina, Sue Bonnington, Lynne Bradshaw, James Catto, Debbie Cooper, Liz Down, Andrew Doble, Alan Doherty, Garrett Durkan, Emma Elliott, David Gillatt, Pippa Herbert, Peter Holding, Joanne Howson, Mandy Jones, Roger Kockelbergh, Rajeev Kumar, Howard Kynaston, Athene Lane, Teresa Lennon, Norma Lyons, Hing Leung, Malcolm Mason, Hilary Moody, Philip Powell, Alan Paul, Stephen Prescott, Derek Rosario, Patricia O'Sullivan, Pauline Thompson, Sarah Tidball.

**Funding for the CRUK study and PRACTICAL consortium**

This work was supported by the Cancer Research UK Grants C5047/A7357, C1287/A10118, C5047/A3354, C5047/A10692, C16913/A6135.

**Funding and Acknowledgements for COGS**

Funding for the iCOGS infrastructure came from: the European Community's Seventh Framework Programme under grant agreement n° 223175 (HEALTH-F2-2009-223175) (COGS), Cancer Research UK (C1287/A10118, C1287/A 10710, C12292/A11174, C1281/A12014, C5047/A8384, C5047/A15007, C5047/A10692, C8197/A16565), the National Institutes of Health (CA128978) and Post-Cancer GWAS initiative (1U19 CA148537, 1U19 CA148065 and 1U19 CA148112 - the GAME-ON initiative), the Department of Defence (W81XWH-10-1-0341), the Canadian Institutes of Health Research (CIHR) for the CIHR Team in Familial Risks of Breast Cancer, Komen Foundation for the Cure, the Breast Cancer Research Foundation, and the Ovarian Cancer Research Fund.

This study would not have been possible without the contributions of the following: Per Hall (COGS); Douglas F. Easton, Paul Pharoah, Kyriaki Michailidou, Manjeet K. Bolla, Qin Wang (BCAC), Andrew Berchuck (OCAC), Rosalind A. Eeles, Douglas F. Easton, Ali Amin Al Olama, Zsofia Kote-Jarai, Sara Benlloch (PRACTICAL), Georgia Chenevix-Trench, Lesley McGuffog, Fergus Couch and Ken Offit (CIMBA), Joe Dennis, Alison M. Dunning, Andrew Lee, and Ed Dicks, Craig Luccarini and the staff of the Centre for Genetic Epidemiology Laboratory, Javier Benitez, Anna Gonzalez-Neira and the staff of the CNIO genotyping unit, Jacques Simard and Daniel C. Tessier, Francois Bacot, Daniel Vincent, Sylvie LaBoissière and Frederic Robidoux and the staff of the McGill University and Génome Québec Innovation Centre, Stig E. Bojesen, Sune F. Nielsen, Borge G. Nordestgaard, and the staff of the Copenhagen DNA laboratory, and Julie M. Cunningham, Sharon A. Windebank, Christopher A. Hilker, Jeffrey Meyer and the staff of Mayo Clinic Genotyping Core Facility

**Additional funding and acknowledgments from studies in PRACTICAL**

The Department of Medical Epidemiology and Biostatistics, Karolinska Institute, Stockholm, Sweden was supported by the Cancer Risk Prediction Center (CRisP; www.crispcenter.org), a Linneus Centre (Contract ID 70867902) financed by the Swedish Research Council, Swedish Research Council (grant no K2010-70X-20430-04-3 and no 2014/2269), the Swedish Cancer Foundation (grant no 09-0677 and no 2012/823), the Hedlund Foundation, the Soederberg Foundation, the Enqvist Foundation, ALF funds from the Stockholm County Council. Stiftelsen Johanna Hagstrand och Sigfrid Linner's Minne, Karlsson's Fund for urological and surgical research. We thank and acknowledge all of the participants in the Stockholm-1 study. We thank Carin Cavalli-Bjoerkman and Ami Roennberg Karlsson for their dedicated work in the collection of data. Michael Broms is acknowledged for his skilful work with the databases. KI Biobank is acknowledged for handling the samples and for DNA extraction. Hans Wallinder at Aleris Medilab and Sven Gustafsson at Karolinska University Laboratory are thanked for their good cooperation in providing historical laboratory results.

The EPIC study acknowledges the following financial support: The coordination of EPIC is financially supported by the European Commission (DG-SANCO) and the International Agency for Research on Cancer. The national cohorts (that recruited male participants) are supported by Danish Cancer Society (Denmark); German Cancer Aid, German Cancer Research Center (DKFZ), Federal Ministry of Education and Research (BMBF), Deutsche Krebshilfe, Deutsches Krebsforschungszentrum and Federal Ministry of Education and Research (Germany); the Hellenic Health Foundation (Greece); Associazione Italiana per la Ricerca sul Cancro-AIRC-Italy and National Research Council (Italy); Dutch Ministry of Public Health, Welfare and Sports (VWS), Netherlands Cancer Registry (NKR), LK Research Funds, Dutch Prevention Funds, Dutch ZON (Zorg Onderzoek Nederland), World Cancer Research Fund (WCRF), Statistics Netherlands (The Netherlands); Health Research Fund (FIS), PI13/00061 to Granada, PI13/01162 to EPIC-Murcia, Regional Governments of Andalucía, Asturias, Basque Country, Murcia and Navarra, ISCIII RETIC (RD06/0020) (Spain); Swedish Cancer Society, Swedish Research Council and County Councils of Skåne and Västerbotten (Sweden); Cancer Research UK (14136 to EPIC-Norfolk; C570/A16491 and C8221/A19170 to EPIC-Oxford), Medical Research Council

(1000143 to EPIC-Norfolk, MR/M012190/1 to EPIC-Oxford) (United Kingdom).

The ESTHER study was supported by a grant from the Baden Württemberg Ministry of Science, Research and Arts. Additional cases were recruited in the context of the VERDI study, which was supported by a grant from the German Cancer Aid (Deutsche Krebshilfe). The ESTHER group would like to thank Hartwig Ziegler, Sonja Wolf, Volker Hermann, Katja Butterbach.

The FHCRC studies were supported by grants RO1CA056678, RO1CA082664, and RO1CA092579 from the US National Cancer Institute, National Institutes of Health, with additional support from the Fred Hutchinson Cancer Research Center.

The IPO-Porto study was in part funded by Liga Portuguesa Contra o Cancro.

The Mayo group was supported by the US National Cancer Institute (R01CA72818).

The Prostate Cancer Program of Cancer Council Victoria also acknowledge grant support from The National Health and Medical Research Council, Australia (126402, 209057, 251533, , 396414, 450104, 504700, 504702, 504715, 623204, 940394, 614296,), VicHealth, Cancer Council Victoria, The Prostate Cancer Foundation of Australia, The Whitten Foundation, PricewaterhouseCoopers, and Tattersall’s.

The MEC was support by NIH grants CA63464, CA54281 and CA098758.

The Moffitt group was supported by the US National Cancer Institute (R01CA128813, PI: J.Y. Park).

The PCMUS study was supported by the Bulgarian National Science Fund, Ministry of Education and Science (contract DOO-119/2009; DUNK01/2-2009; DFNI-B01/28/2012) with additional support from the Science Fund of Medical University - Sofia (contract 51/2009; 8I/2009; 28/2010).

ProtecT would like to acknowledge the support of The University of Cambridge, Cancer Research UK. Cancer Research UK grants [C8197/A10123] and [C8197/A10865] supported the genotyping team. We would also like to acknowledge the support of the National Institute for Health Research which funds the Cambridge Bio-medical Research Centre, Cambridge, UK. We would also like to acknowledge the support of the National Cancer Research Prostate Cancer: Mechanisms of Progression and Treatment (PROMPT) collaborative (grant code G0500966/75466) which has funded tissue and urine collections in Cambridge. We are grateful to staff at the Welcome Trust Clinical Research Facility, Addenbrooke’s Clinical Research Centre, Cambridge, UK for their help in conducting the ProtecT study. We also acknowledge the support of the NIHR Cambridge Biomedical Research Centre, the DOH HTA (ProtecT grant) and the NCRI / MRC (ProMPT grant) for help with the bio-repository. The UK Department of Health funded the ProtecT study through the NIHR Health Technology Assessment Programme (projects 96/20/06, 96/20/99). The ProtecT trial and its linked ProMPT and CAP (Comparison Arm for ProtecT) studies are supported by Department of Health, England; Cancer Research UK grant number C522/A8649, Medical Research Council of England grant number G0500966, ID 75466 and The NCRI, UK. The epidemiological data for ProtecT were generated though funding from the Southwest National Health Service Research and Development. DNA extraction in ProtecT was supported by USA Dept of Defense award W81XWH-04-1-0280, Yorkshire Cancer Research and Cancer Research UK. The authors would like to acknowledge the contribution of all members of the ProtecT study research group. The views and opinions expressed therein are those of the authors and do not necessarily reflect those of the Department of Health of England. The bio-repository from ProtecT is supported by the NCRI (ProMPT) Prostate Cancer Collaborative and the Cambridge BMRC grant from NIHR.

The QLD research is supported by The National Health and Medical Research Council, Australia Project Grant [390130, 1009458] and Enabling Grant [614296 to APCB]; the Prostate Cancer Foundation of Australia (Project Grant [PG7] and Research infrastructure grant [to APCB]). Australian Prostate Cancer BioResource acknowledges the Queensland node participants in this study including– Trina Yeadon, Pamela Saunders, Allison Eckert and Judith Clements - Institute of Health and Biomedical Innovation, Queensland University of Technology, Brisbane, Queensland, Australia; Peter Heathcote, Glenn Wood, Greg Malone - Brisbane Urology Clinic, Wickham Terrace, Brisbane, Qld, Australia; Hema Samaratunga - Aquesta Pathology, Toowong, QLD, Australia; Angus Collins, Megan Turner and Kris Kerr - Sullivan and Nicolaides Pathology, Brisbane, Qld, Australia

SCCS is funded by NIH grant R01 CA092447, and SCCS sample preparation was conducted at the Epidemiology Biospecimen Core Lab that is supported in part by the Vanderbilt-Ingram Cancer Center (P30 CA68485). Data on SCCS cancer cases used in this publication were provided by the Alabama Statewide Cancer Registry; Kentucky Cancer Registry, Lexington, KY; Tennessee Department of Health, Office of Cancer Surveillance; Florida Cancer Data System; North Carolina Central Cancer Registry, North Carolina Division of Public Health; Georgia Comprehensive Cancer Registry; Louisiana Tumor Registry; Mississippi Cancer Registry; South Carolina Central Cancer Registry; Virginia Department of Health, Virginia Cancer Registry; Arkansas Department of Health, Cancer Registry, 4815 W. Markham, Little Rock, AR 72205. The Arkansas Central Cancer Registry is fully funded by a grant from National Program of Cancer Registries, Centers for Disease Control and Prevention (CDC). Data on SCCS cancer cases from Mississippi were collected by the Mississippi Cancer Registry which participates in the National Program of Cancer Registries (NPCR) of the Centers for Disease Control and Prevention (CDC). The contents of this publication are solely the responsibility of the authors and do not necessarily represent the official views of the CDC or the Mississippi Cancer Registry.

SEARCH is funded by a programme grant from Cancer Research UK [C490/A10124] and supported by the UK National Institute for Health Research Biomedical Research Centre at the University of Cambridge.

The Tampere (Finland) study was supported by the Academy of Finland (251074), The Finnish Cancer Organisations, Sigrid Juselius Foundation, and the Competitive Research Funding of the Tampere University Hospital (9N069 and X51003). The PSA screening samples were collected by the Finnish part of ERSPC (European Study of Screening for Prostate Cancer). Riina Liikanen is thanked for technical assistance. Riitta Vaalavuo and Liisa Maeaettaenen are thanked for their work with databases.

UKGPCS would also like to thank the following for funding support: The Institute of Cancer Research and The Everyman Campaign, The Prostate Cancer Research Foundation, Prostate Research Campaign UK (now Prostate Action), The Orchid Cancer Appeal, The National Cancer Research Network UK, The National Cancer Research Institute (NCRI) UK. We are grateful for support of NIHR funding to the NIHR Biomedical Research Centre at The Institute of Cancer Research and The Royal Marsden NHS Foundation Trust. UKGPCS should also like to acknowledge the NCRN nurses, data managers and Consultants for their work in the UKGPCS study. UKGPCS would like to thank all urologists and other persons involved in the planning, coordination, and data collection of the CAPS study.

The Ulm group received funds from the German Cancer Aid (Deutsche Krebshilfe).

The Keith and Susan Warshaw Fund, C. S. Watkins Urologic Cancer Fund and The Tennity Family Fund supported the Utah study. The project was supported by Award Number P30CA042014 from the National Cancer Institute.
