# Supplementary material for: Gene and pathway level analyses of germline DNA-repair gene variants and prostate cancer susceptibility using the iCOGS-genotyping array
Source: Br J Cancer. 2016 Mar 10;114(8):945–52. doi: 10.1038/bjc.2016.50 (PMC5379914; doi:10.1038/bjc.2016.50)
Supplement: Supplementary Table 2 [file bjc201650x7.docx]

|  | **All Variants** | | | **Typed** | | | **Imputed** | | |
| --- | --- | --- | --- | --- | --- | --- | --- | --- | --- |
|  | **Del** | **Ins** | **SNP** | **Del** | **Ins** | **SNP** | **Del** | **Ins** | **SNP** |
| **Coding** | 44 | 32 | 1900 | 0 | 0 | 218 | 44 | 32 | 1682 |
| **UTR** | 140 | 94 | 1717 | 0 | 0 | 158 | 140 | 94 | 1559 |
| **Splice** | 1 | 3 | 12 | 0 | 0 | 2 | 1 | 3 | 10 |
| **Intronic** | 3079 | 2246 | 39510 | 0 | 0 | 2025 | 3079 | 2246 | 37485 |
| **Intergenic** | 2312 | 1539 | 28674 | 0 | 0 | 1107 | 2312 | 1539 | 27567 |
