# Supplementary material for: Gene and pathway level analyses of germline DNA-repair gene variants and prostate cancer susceptibility using the iCOGS-genotyping array
Source: Br J Cancer. 2016 Mar 10;114(8):945–52. doi: 10.1038/bjc.2016.50 (PMC5379914; doi:10.1038/bjc.2016.50)
Supplement: Supplementary Information [file bjc201650x11.docx]

**Supplementary Note – List of additional authors, funding information and acknowledgements for consortia and individual studies.**

**Supplementary Table 1 – List of the 179 DNA repair genes analysed in this study.** Indicated are the boundaries of the region analysed for each gene, the number of variants within each region available for analysis after QC and typed variants only and the main DNA repair pathway that each gene participates in.

**Supplementary Figure 1 – (a)** MAF distribution of the variants analysed in this study. Rare and uncommon variants represented a substantial proportion of the dataset.

Barplots showing classification and relative frequencies of the variants analysed in this study for **(b)** all variants analysed **(c)** typed variants **(d)** imputed variants. For each classification, the proportion of single nucleotide polymorphisms within the dataset is denoted in blue, insertion variants in green and deletions in red.

**Supplementary Table 2 – Number of variants within each classification group.** Variants were annotated using wANNOVAR and categorised into five groups; coding, UTR, splice, intronic and intergenic. Numbers within each group are shown for the total set analysed and typed and imputed variants separately.

**Figure 1 - Single SNP Case-Control Manhattan Plot.** 81,303 SNPs from 179 DNA repair genes were analysed for association with PrCa. Only the previously reported association within the *RAD51B* gene was identified, with suggestive, non-significant association peaks observed at a small number of other loci.

**Supplementary Table 3 – Case-Control single SNP association data (.xlsx file). Analyses were performed on 21,780 PrCa cases and 21,727 controls, with per allele odds ratios estimated by logistic regression.**

**Supplementary Figure 2 – QQ plots for all the DNA repair gene variants analysed. (a)** All SNPs analysed **(b)** All SNPs with the *RAD51B* region excluded.

Modest genomic inflation was observed within the DNA repair gene dataset (λ = 1.105) after adjusting for the first 8 principal components, however deviation from the null was still observed towards the extremity of the observed distributions. This was predominantly explained by the association with *RAD51B*, although the null was not fully recovered even with this region removed.

**Figure 2 – Case-Control Manhattan Plots for the 179 DNA repair genes analysed by SKAT. (a)** A significant association was observed for the *MSH5* gene using the SKAT-C test that examines the combined effect of common and rare variants. **(b)** No significant association was detected for any gene under the SKAT-O test that primarily focuses on rare variant association testing.

**Supplementary Table 4 – Case-Control gene and pathway level association data (.xlsx file). Results for gene, pathway and pathway (coding variants only) level analyses are shown in separate worksheets. The Worksheet Legend sheet contains additional details of the phenotypes, tests performed, sample numbers and significance thresholds for each worksheet.**

**Supplementary Figure 3 – Manhattan Plots for Case-Case analyses of DNA repair gene variants associated with more aggressive PrCa clinical presentation. (a)** Single SNP analysis of NCCN1 vs. M^+^/N^+^ patients **(b)** Single SNP analysis of Gleason ≤6 vs. Gleason ≥8 patients **(c)** SKAT-C gene level analysis of NCCN1 vs. M^+^/N^+^ patients **(d)** SKAT-C gene level analysis of Gleason ≤6 vs. Gleason ≥8 patients **(e)** SKAT-O gene level analysis of NCCN1 vs. M^+^/N^+^ patients **(f)** SKAT-O gene level analysis of Gleason ≤6 vs. Gleason ≥8 patients.

**Supplementary Table 5 – Case-Case single SNP and gene level association data (.xlsx file). Results for single SNP, gene, pathway and pathway (coding variants only) level tests for both phenotype criteria analysed are shown in separate worksheets. The Worksheet Legend sheet contains additional details of the phenotypes, tests performed, sample numbers and significance thresholds for each worksheet.**

**Supplementary Figure 4 – Locus Explorer visualisation of the *RAD23B* locus at Chr9q31.** The cluster of variants in purple indicate the variants analysed in this study for the *RAD23B* gene and 20kb flanking region, whilst the position of the previously reported PrCa risk signals in the Chinese (rs817826) and European (index SNP rs1771718) populations are plotted in green and yellow respectively (*P*-values shown on the plot are from the original studies and therefore the three individual signals are not directly comparable for statistical data). These previously reported risk variants are situated >50kb from the *RAD23B* gene, well outside of the region analysed in this study, and cluster around a regulatory element in the LNCaP cell-line. No variants within the gene centric window analysed in this study were significant after adjustment for multiple testing. Image generated using Locus Explorer (Dadaev *et al*, in press).
